# Multimorbidity patterns and influencing factors in older Chinese adults: a national population-based cross-sectional survey

**DOI:** 10.7189/jogh.15.04051

**Published:** 2025-02-21

**Authors:** Xinyu Xue, Ziyi Wang, Yana Qi, Ningsu Chen, Kai Zhao, Mengnan Zhao, Lei Shi, Jiajie Yu

**Affiliations:** 1 Department of Clinical Nutrition, West China Hospital, Sichuan University, Chengdu, China; 2 Chinese Evidence-based Medicine Center, West China Hospital, Sichuan University, Chengdu, China; 3 Department of Hematology, West China Hospital, Sichuan University, Chengdu, China; 4 Department of Electric Information, Sichuan University, Chengdu, China

## Abstract

**Background:**

This study aims to develop specific multimorbidity relationships among the elderly and to explore the association of multidimensional factors with these relationships, thereby facilitating the formulation of personalised strategies for multimorbidity management.

**Methods:**

Cluster analysis identified chronic conditions that tend to cluster together, and then association rule mining was used to investigate relationships within these identified clusters more closely. Stepwise logistic regression analysis was conducted to explore the relationship between influencing factors and different health statuses in older adults. The results of this study were presented by network graph visualisation.

**Results:**

A total of 15 045 individuals were included in this study. The average age was 73.0 ± 6.8 years. The number of patients with multimorbidity was 7426 (49.4%). The most common binary disease combination was hypertension and depression. The four major multimorbidity clusters identified were the tumour-digestive disease cluster, the metabolic-circulatory disease cluster, the metal-psychological disease cluster, and the age-related degenerative disease cluster. Cluster analysis by sex and region revealed similar numbers and types of conditions in each cluster, with some variations. Gender and number of medications had a consistent effect across all disease clusters, while aging, body mass index (BMI), waist-to-hip ratio (WHR), cognitive impairment, plant-based foods, animal-based foods, highly processed foods and marital status had varying effects across different disease clusters.

**Conclusions:**

Multimorbidity is highly prevalent in the older population. The impact of lifestyle varies between different clusters of multimorbidity, and there is a need to implement different strategies according to different clusters of multimorbidity rather than an integrated approach to multimorbidity management.

The elderly population in China is projected to reach approximately 4.3 billion (16%) by 2050, reflecting a rapid and widespread aging trend [1,2]. This shift is accompanied by an increase in chronic diseases, with individuals more likely to have multiple chronic conditions [3–5]. Multiple chronic conditions, also known as multimorbidity, defined as the co-occurrence of two or more chronic conditions within an individual, are expected to become increasingly common [6]. The prevalence of two or more conditions in people over 65 is estimated to rise globally from 54% in 2015 to 68% in 2035[7]. Furthermore, the number of people with four or more conditions is projected to double by 2035, reaching almost 17% [7].

Multimorbidity leads to increased mortality and disability [8,9], reduced physical functioning, lower quality of life and poorer self-rated health [10], posing a heavy medical burden on the health care system [11,12]. The treatment of multiple chronic conditions primarily relies on single-disease strategies [13]. However, multimorbidity management requires a fundamentally different approach. While traditional management begins by defining a specific disease, multimorbidity management prioritises defining the population, exploring health determinants, and understanding how these factors vary across different groups [14]. This shift necessitates moving away from single-disease strategies and adopting approaches that consider the complex interplay of multiple conditions within individuals [15].

Patients with multimorbidity may have specific interrelationships between their conditions. Identifying these relationships is crucial for clinicians to predict disease progression and develop effective interventions. We conducted a scoping review to explore the multimorbidity patterns in China and found that most studies in the review were limited by narrow definitions of multimorbidity, often including only a small number of chronic conditions (manuscript under review). Meanwhile, these studies frequently focus on physical illnesses while excluding psychiatric disorders like depression and anxiety, potentially underestimating the true prevalence and impact of multimorbidity [16–19].

The pathogenesis of multimorbidity is complex, involving biological, behavioural, and societal factors [20–22]. Our previous scoping review found that most studies focus on individual factors such as age, gender, smoking, and geographic environment. While important, these factors alone do not fully capture the multidimensional nature of multimorbidity (manuscript under review). Increasingly, research adopts biopsychosocial and health-ecological perspectives, emphasising the interplay of multiple factors in driving chronic disease and multimorbidity [23–26]. However, these studies often fail to explore how specific factors interact within multimorbidity relationships [27–29], limiting a comprehensive understanding of how conditions influence each other in various contexts, which is essential for adapting interventions and more accurately predicting patient outcomes. Additionally, common methods like exploratory factor analysis (EFA) [30], latent class analysis (LCA) [31], and clustering [32] identify broad patterns but do not clarify the associations between individual conditions within these patterns. New approaches are needed to better assess multimorbidity patterns in affected populations.

To address the current research gaps, improve our understanding of factors influencing multimorbidity, and provide more targeted and personalised management strategies, we conducted a cross-sectional study utilising data from the 2018 China Longitudinal Healthy Longevity Survey (CLHLS). Our study aims to 1) examine the prevalence of multimorbidity and multimorbidity clusters in Chinese older adults aged 65 years and above; 2) explore the biological, psychological, social, and lifestyle factors with specific multimorbidity; and 3) construct multimorbidity patterns combining multimorbidity clusters and their influencing factors.

## METHODS

### Study participants

Since the Chinese Longitudinal Healthy Longevity Survey (CLHLS) questionnaire did not include lifestyle data, such as medication and nutritional supplement use, before 2015, we used data from the 2017–2018 wave of the CLHLS to ensure a more comprehensive analysis. The CLHLS is an ongoing longitudinal study that began in 1998 and aims to identify the determinants of healthy aging and longevity in the older population (≥65 years) in China. Trained interviewers conducted in-home surveys using a structured questionnaire. Details on the sampling procedure and assessment of data quality can be found in previous publications. The CLHLS was approved by the Biomedical Ethics Committee, Peking University (IRB00001052–13074). All participants provided written informed consent prior to participation.

The 2017–2018 wave of CLHLS interviewed 15 874 individuals aged 65 and older. We included community-dwelling participants aged 65 and older with demographic data. Exclusion criteria were individuals with hearing impairment who completed the questionnaire on themselves, those with incomplete disease data, those with incomplete data on depression and anxiety scales, and those with more than 20% of incomplete data.

### Data collection

The 2017–2018 wave of CLHLS collected data using standardised questionnaires covering demographic characteristics, family and household characteristics, personality and emotional traits, lifestyle, diet, self-reported health, lower and upper extremity function and chronic diseases. For participants with disabilities who were unable to answer the questions, their primary family carers were interviewed as proxy respondents. Systematic assessments of the CLHLS for reliability, validity, and consistency of various measures, as well as for attrition, demonstrated good data quality.

### Outcome variable

We adopted the most widely used deﬁnition of multimorbidity, which is the coexistence of more than two chronic conditions in the same individual. The CLHLS included a comprehensive list of 22 chronic conditions: hypertension, glaucoma, cancer, stroke and cerebrovascular diseases, chronic lung disease, heart disease, cataracts, prostate disease, diabetes, chronic gastrointestinal ulcers, pressure sores, arthritis, Parkinson disease, dementia, epilepsy, dyslipidemia, rheumatism or rheumatoid arthritis, chronic cholecystitis or cholelithiasis, chronic nephritis, uterine fibroids, chronic hepatitis, mammary gland hyperplasia. Participants also could report other chronic diseases not explicitly listed in the questionnaire. The presence of these conditions was self-reported by the respondents and confirmed by hospital diagnosis. Given the critical importance of depression and anxiety to the health of older adults, and recognising the long-standing neglect of psychiatric disorders in multimorbidity studies, we included depression and anxiety disorders with the 22 conditions listed above.

The 10-item Center for Epidemiologic Studies Depression Scale (CES-D-10) was employed to measure depressive symptoms, which consists of ten questions, each of which is divided into five levels of scoring. For the negative state questions and sleep quality questions, responses were scored as follows: ‘rarely or never’ as 0, ‘sometimes’ as 1, ‘often’ as 2, and ‘always’ as 3. Positive state questions were scored in reverse. The scale has a theoretical score range of 0–30, and in this study a score of ten indicated depression.

The 7-item Generalized Anxiety Disorder Scale (GAD-7) was employed to measure anxiety symptoms, which consists of seven questions, each with four scoring levels. Responses of ‘never’, ‘occasionally’, ‘over half the time’, ‘almost daily’ were scored 0, 1, 2, and 3, respectively. The scale has a theoretical score range of 0 to 21, and a score of five indicates anxiety. The Chinese language questionnaires used in the CLHLS have recently gained wide acceptance and use [18].

### Independent variable

To identify the independent variables for this study, we used the 'Petal' theoretical framework and the findings from our scoping review to determine the factors that may influence multimorbidity. The ‘Petal’ framework is a conceptual model that visualises the multifactorial determinants of multimorbidity as interconnected ‘petals’ of a flower, emphasising the complex interplay between biological, behavioural, social, and environmental factors. Guided by this framework, we categorised the independent variables into three domains: personal characteristics (age, gender, body mass index, waist-to-hip ratio, cognitive impairment), lifestyle characteristics (drinking smoking, physical activity, dietary pattern, nutritional supplement and number of medications), and socioeconomic characteristics (residential status, marital status, public insurance, education, residential area). We categorised provinces into four regions: Eastern Region (Shanghai, Jiangsu, Zhejiang, Anhui, Fujian, Jiangxi and Shandong); Southern Region (Guangdong, Guangxi, Hainan, Chongqing, and Sichuan), Central Region (Henan, Hubei and Hunan), Northern Region (Beijing, Tianjin, Hebei, Shanxi, Liaoning, Jilin, Heilongjiang and Shaanxi).

#### Cognitive impairment assessment

The CLHLS used the Chinese version of the Mini-mental State Examination (MMSE), which have been shown to have validity and reliability, as a measure of cognitive function at each wave [33–35]. The MMSE consists of 30 items evaluating orientation, registration, attention and calculation, recall, and language. Scores range from 0 to 30, with higher scores indicating better cognitive function. We used education-adjusted criteria to define ‘cognitive impairment (CI)’: for participants with no formal education, MMSE score ≤17 was defined as CI; for those with 1–6-year education, MMSE score ≤20 was defined as CI; for those with more than 6-year education, MMSE score ≤24 was defined as CI. According to this criterion, the presence of cognitive impairment is scored as 1 and vice versa as 0.

#### Dietary pattern assessment

We assessed dietary patterns using a method from Anna Zhu et al., which included 16 food groups representing the most common foods in the Chinese daily diet [36–38]. These food groups were categorised into three types: plant-based foods, highly processed foods and animal-based foods. Plant-based foods included whole grains, vegetable oils, fruits, vegetables, garlic, soy products, nuts, and tea. Highly processed foods consisted of refined grains, pickled vegetables or kimchi, and sugars. Animal-based foods included animal fats, meat, seafood, eggs, and dairy products. Each food was scored on a scale from 1 to 5. The total score for plant-based foods ranged from 8 to 40, for highly processed foods ranged from 3 to 15, and for animal-based foods ranged from 5 to 25. Details on specific foods and their scoring criteria were provided in the Appendix S1 in the **Online Supplementary Document**.

### Data for weighted adjustment

To avoid the problem of small sub-sample sizes in the more advanced ages, the CLHLS interviewed almost all centenarians and over-sampled the oldest-old of more advanced ages, especially among males. Therefore, to ensure that the sample was nationally representative, we used a complex sampling weighting method to adjust for the study population [39].

The specific weight w(x, s, r, t) for age (x), sex (s), and rural-urban residence (r) in the survey year t is computed as follows:



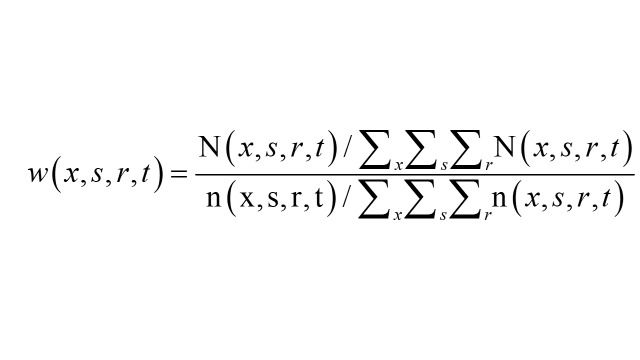





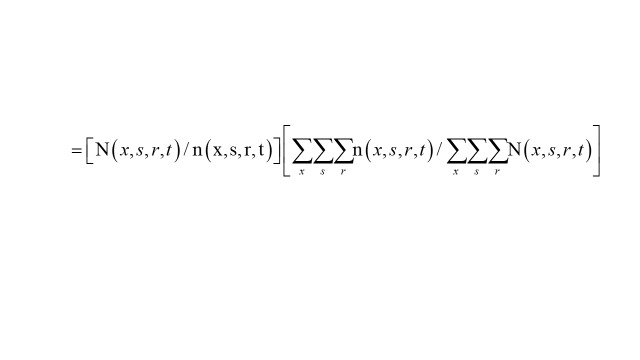



N(x, s, r, t) is the number of persons of age x, sex s and residence r in year t, derived from the predicted elderly population based on the most recent census and the estimated age-sex-specific survival probabilities between the census year and survey year t for the 22 counties in which the CLHLS survey was conducted. (x, s, r, t) is the number of persons of age x, sex s, and residence r as observed in the CLHLS survey conducted in the year t. The weight w(x, s, r, t) is the ratio of the age distribution of the entire elderly population in the survey year t to the age distribution of the sample in the year t.

### Statistical analysis

Categorical variables were presented as numbers and percentages, while continuous variables were expressed as mean (and standard deviation) or median (and range). We used the Pearson χ^2^ trend test or the Wilcoxon Rank Sum Test to compare the prevalence of multimorbidity among the elderly with diverse chronic disease types and demographic characteristics. All statistical tests were two-sided, and a *P*-value of 0.05 or lower was considered statistically significant. The multiple imputation (MI) method used to deal with missing values of continuous independent variables. The mode or median was used to handle missing values of categorical independent variables [40].

We used cluster analysis [41] to identify chronic conditions that tend to cluster together and then used association rule mining (ARM) to investigate relationships within these identified clusters in more details [42]. Cluster analysis was performed using the hierarchical clustering algorithm. Class averaging was used to determine the number of categories in the clusters during the clustering process and tested for clinical significance. Yule's Q distance was used as a similarity calculation method to measure the dissimilarity between chronic diseases. Since the number of diseases contained in different clusters varied, we used different settings: the minimum conditional support was set to 1%, the maximum number of antecedents was set to 2, and the minimum confidence levels were set to 50, 30, 10, 5, and 1%, in that order, and these confidence levels were explored from highest to lowest until the rules were found. Three commonly used metrics were used: support (how often the disease combinations occur in the data set), conﬁdence (the conditional probability that a participant with the antecedent disease will also have the consequence disease), and lift (the ratio of the observed support to that expected if the two events were independent). Lift measures the importance of a rule within ARM and was therefore considered the main measure of significance in the study. The higher the lift, the higher the probability of the consequent co-occurring with the antecedent and the more significant the association [43]. The results of ARM analyses are presented using summary tables of association rules and graphical visualisations of disease combinations. Variations of multimorbidity relationships were assessed by sex and regions.

The health status was categorised into three groups: healthy, those with only one disease, and those with multimorbidity. If the ARM did not identify specific multimorbidity relationships with a lift greater than 1 within the clusters formed by cluster analysis, regression analysis was not performed. Stepwise logistic regression analysis was conducted with health status as the dependent variable and various influencing factors as independent variables to investigate the association.

We used a bimodal network to reveal the relationships between chronic diseases and their influencing factors. In the network graph, circles (or ‘nodes’) represented different chronic diseases, with the size of each circle reflecting the disease's prevalence. The lines connecting these circles indicated correlations between different chronic diseases, and the thickness of these lines represented the strength of the multimorbidity association – thicker lines signified stronger associations. Solid lines indicated associations confirmed by ARM, while dashed lines suggested that, although no associations with a lift greater than 1 were found, the diseases belonged to the same cluster in the clustering analysis. Triangular nodes represented different influencing factors, with light colors indicating statistical significance in comparisons between participants with one chronic disease and healthy participants, and dark colors indicating statistical significance in comparisons between the multimorbidity group and the healthy group. Green represented protective factors, while red represented risk factors. The lines between nodes showed the relationships between influencing factors and chronic diseases. Furthermore, each edge in the network graph was assigned a weight and the edges were undirected, forming an undirected weighted network.

We used IBM SPSS version 29.0 (IBM Corporation, Armonk, New York, USA, 2023), SPSS Modeler 18.0 (IBM Corporation, Armonk, New York, USA, 2017), STATA 16 (StataCorp LLC, College Station, Texas, USA, 2019) and Cytoscape version 3.9.1 (Cytoscape Consortium, San Diego, California, USA, 2021) to conduct the statistical analysis.

## RESULTS

### Participant characteristics

**Figure 1:**
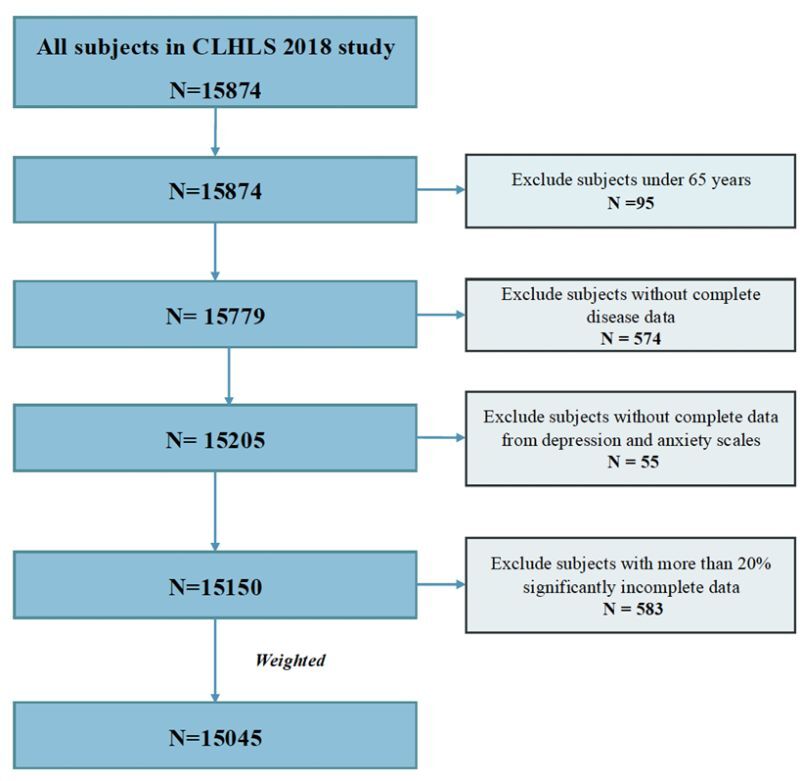
Flowchart showing the selection of participants.

A total of 15 045 participants were included in this study (**Figure 1**), of which 47.9% were male and 52.1% were female, with a mean age of 73.0 years. Among these elderly people, 4235 (28.1%) had one chronic disease, 3108 (20.7%) had two chronic diseases, and 7426 (49.4%) had multimorbidity. There were significant differences in all characteristics between the three groups except for nuts consumption and education level. Hypertension, depression and heart disease were the three most common conditions, with prevalence of 44.6%, 24.9%, and 16.1%, respectively (Appendix S2 in the **Online Supplementary Document**).

The mean score for plant-based foods, highly processed foods and animal-based foods were 24.1, 9.6 and 14.9, respectively. Among the participants, 4468 (29.7%) consumed alcohol, 5010 (33.3%) smoked, and 13555 (90.1%) were physically active. More than half (59.5%) were currently taking medication, while only 1728 (11.5%) used nutritional supplements. Additionally, 8649 (57.5%) were covered by public old age insurance, 7276 (48.3%) had primary education, and 7434 (50.0%) lived in rural areas. The characteristics of study participants are shown in **Table 1**.

**Table 1:** Characteristics of all participants

Characteristics	Total, n = 15 045 (%)	Healthy n = 3383 (%)	One disease n = 4236 (%)	Multimorbidity n = 7426 (%)	*P*-value*
**Gender**					<0.001
Male	7212 (47.9)	1857 (54.9)	2139 (50.5)	3215 (43.3)	
Female	7833 (52.1)	1526 (45.1)	2097 (49.5)	4211 (56.7)	
**Age, year**					
x̄ ± SD	73.0 ± 6.8	72.1 ± 6.6	72.8 ± 6.8	73.6 ± 6.9	<0.001†
**Ethnicity**					<0.001
Han	14 058 (93.4)	3035 (89.7)	3939 (93.0)	7084 (95.4)	
Other	987 (6.6)	348 (10.3)	297 (7.0)	342 (4.6)	
**Residential status**					<0.001
Living alone	2029 (13.5)	386 (11.4)	559 (13.2)	1084 (14.6)	
Living with others	13 016 (86.5)	2997 (88.6)	3677 (86.8)	6342 (85.4)	
**Marital status**					<0.001
Currently married	10 623 (70.6)	2524 (74.6)	3080 (72.7)	5020 (67.6)	
Divorced	4256 (28.3)	832 (24.6)	1114 (26.3)	2309 (31.1)	
Never married	166 (1.1)	27 (0.8)	42 (1.0)	97 (1.3)	
**Heart rate, in beats/min**					
x̄ ± SD	75.2 ± 10.1	74.7 ± 9.2	75.3 ± 9.9	75.4 ± 10.5	0.003†
**Weigh, kg**					
x̄ ± SD	59.1 ± 12.1	58.2 ± 11.6	58.7 ± 12.1	59.8 ± 12.3	<0.001†
**Height, cm**					
x̄ ± SD	157.9 ± 9.7	158.5 ± 9.3	157.9 ± 10.0	157.6 ± 9.6	<0.001†
**Waist circumference, cm**					
x̄ ± SD	86.6 ± 10.4	85.3 ± 9.9	86.1 ± 10.2	87.6 ± 10.6	<0.001†
**Hip circumference, cm**					
x̄ ± SD	94.3 ± 9.2	93.2 ± 8.8	93.7 ± 8.8	95.2 ± 9.6	<0.001†
**BMI, kg/m^2^**					
x̄ ± SD	23.7 ± 5.8	23.1 ± 4.7	23.6 ± 6.4	24.1 ± 5.9	<0.001†
**WHR**					
x̄ ± SD	0.9 ± 0.1	0.9 ± 0.1	0.9 ± 0.1	0.9 ± 0.1	<0.001†
**Systolic, mmHg**					
x̄ ± SD	138.9 ± 18.8	134.8 ± 16.9	139.8 ± 19.2	140.3 ± 19.1	<0.001†
**Diastolic, mmHg**					
x̄ ± SD	80.8 ± 10.2	79.5 ± 9.2	81.3 ± 10.5	81.1 ± 10.4	<0.001†
**Staple food**					<0.001
Rice	8996 (59.8)	1999 (59.1)	2533 (59.8)	4463 (60.1)	
Mixed grains	583 (3.9)	105 (3.1)	114 (2.7)	364 (4.9)	
Flour	2730 (18.1)	666 (19.7)	839 (19.8)	1225 (16.5)	
Rice and flour	2676 (17.8)	599 (17.7)	733 (17.3)	1344 (18.1)	
Other	60 (0.4)	14 (0.4)	17 (0.4)	30 (0.4)	
**Types of common cooking oil**					<0.001
Vegetable oil	13 607 (90.4)	3011 (89.0)	3817 (90.1)	6780 (91.3)	
Sesame oil	83 (0.5)	17 (0.5)	21 (0.5)	45 (0.6)	
Lard	1279 (8.5)	341 (10.1)	373 (8.8)	564 (7.6)	
Animal fat	76 (0.5)	14 (0.4)	25 (0.6)	37 (0.5)	
**Drinking**	4468 (29.7)	1086 (32.1)	1347 (31.8)	2035 (27.4)	<0.001
**Smoking**	5010 (33.3)	1204 (35.6)	1474 (34.8)	2332 (31.4)	<0.001
**Physical exercise**	13 555 (90.1)	3112 (92.0)	3893 (91.9)	6550 (88.2)	<0.001
**Vegetables**					<0.001
Almost every day	10 394 (69.1)	2378 (70.3)	2974 (70.2)	5042 (67.9)	
Often	3667 (24.4)	836 (24.7)	1012 (23.9)	1819 (24.5)	
Sometimes	766 (5.1)	125 (3.7)	195 (4.6)	446 (6.0)	
Rarely or never	218 (1.4)	44 (1.3)	55 (1.3)	119 (1.6)	
**Fruits**					<0.001
Almost every day	3400 (22.6)	795 (23.5)	979 (23.1)	1626 (21.9)	
Often	3898 (25.9)	1055 (31.2)	1067 (25.2)	1775 (23.9)	
Sometimes	4650 (30.9)	978 (28.9)	1356 (32.0)	2317 (31.2)	
Rarely or never	3097 (20.6)	555 (16.4)	834 (19.7)	1708 (23.0)	
**Meat**					<0.001
Almost every day	5966 (39.7)	1499 (44.3)	1682 (39.7)	2785 (37.5)	
≥1 time/week	5956 (39.6)	1326 (39.2)	1682 (39.7)	2948 (39.7)	
≥1 time/mo	1447 (9.6)	264 (7.8)	381 (9.0)	802 (10.8)	
Sometimes	631 (4.2)	135 (4.0)	199 (4.7)	297 (4.0)	
Rarely or never	1045 (6.9)	159 (4.7)	292 (6.9)	594 (8.0)	
**Aquatic products**					<0.001
Almost every day	1488 (9.9)	332 (9.8)	407 (9.6)	750 (10.1)	
≥1 time/week	6194 (41.2)	1391 (41.1)	1766 (41.7)	3037 (40.9)	
≥1 time/mo	3073 (20.4)	754 (22.3)	826 (19.5)	1493 (20.1)	
Sometimes	1895 (12.6)	487 (14.4)	517 (12.2)	891 (12.0)	
Rarely or never	2395 (15.9)	419 (12.4)	720 (17.0)	1255 (16.9)	
**Eggs**					<0.001
Almost every day	5209 (34.6)	1198 (35.4)	1428 (33.7)	2584 (34.8)	
≥1 time/week	5690 (37.8)	1313 (38.8)	1622 (38.3)	2755 (37.1)	
≥1 time/mo	1762 (11.7)	408 (12.1)	521 (12.3)	832 (11.2)	
Sometimes	1000 (6.6)	247 (7.3)	292 (6.9)	460 (6.2)	
Rarely or never	1384 (9.2)	217 (6.4)	373 (8.8)	795 (10.7)	
**Soy products**					<0.001
Almost every day	1821 (12.1)	406 (12.0)	517 (12.2)	899 (12.1)	
≥1 time/week	5924 (39.4)	1360 (40.2)	1711 (40.4)	2852 (38.4)	
≥1 time/mo	3238 (21.5)	792 (23.4)	932 (22.0)	1515 (20.4)	
Sometimes	2038 (13.5)	470 (13.9)	551 (13.0)	1017 (13.7)	
Rarely or never	2024 (13.5)	355 (10.5)	525 (12.4)	1144 (15.4)	
**Pickled vegetables or kimchi**					0.003
Almost every day	2176 (14.5)	447 (13.2)	593 (14.0)	1136 (15.3)	
≥1 time/week	2766 (18.4)	653 (19.3)	754 (17.9)	1359 (18.3)	
≥1 time/mo	1908 (12.7)	440 (13.0)	555 (13.1)	913 (12.3)	
Sometimes	2807 (18.7)	687 (20.3)	754 (17.8)	1366 (18.4)	
Rarely or never	5388 (35.8)	1156 (34.2)	1580 (37.3)	2651 (35.7)	
**Sugars**					<0.001
Almost every day	1198 (8.0)	298 (8.8)	343 (8.1)	557 (7.5)	
≥1 time/week	2433 (16.2)	612 (18.1)	669 (15.8)	1151 (15.5)	
≥1 time/mo	1930 (12.8)	433 (12.8)	487 (11.5)	1010 (13.6)	
Sometimes	2762 (18.4)	660 (19.5)	877 (20.7)	1225 (16.5)	
Rarely or never	6722 (44.7)	1380 (40.8)	1860 (43.9)	3483 (46.9)	
**Garlic**					<0.001
Almost every day	3673 (24.4)	771 (22.8)	1067 (25.2)	1834 (24.7)	
≥1 time/week	3974 (26.4)	995 (29.4)	1131 (26.7)	1849 (24.9)	
≥1 time/mo	2424 (16.1)	514 (15.2)	729 (17.2)	1181 (15.9)	
Sometimes	2310 (15.4)	504 (14.9)	551 (13.0)	1255 (16.9)	
Rarely or never	2664 (17.7)	599 (17.7)	758 (17.9)	1307 (17.6)	
**Dairy products**					<0.001
Almost every day	2953 (19.6)	541 (16.0)	771 (18.2)	1641 (22.1)	
≥1 time/week	2267 (15.1)	477 (14.1)	602 (14.2)	1188 (16.0)	
≥1 time/mo	1487 (9.9)	335 (9.9)	373 (8.8)	780 (10.5)	
Sometimes	1987 (13.2)	491 (14.5)	665 (15.7)	832 (11.2)	
Rarely or never	6351 (42.2)	1539 (45.5)	1826 (43.1)	2985 (40.2)	
**Nuts**					0.239
Almost every day	1170 (7.8)	244 (7.2)	347 (8.2)	579 (7.8)	
≥1 time/week	2383 (15.8)	535 (15.8)	661 (15.6)	1188 (16.0)	
≥1 time/mo	2019 (13.4)	476 (14.1)	546 (12.9)	995 (13.4)	
Sometimes	3182 (21.2)	758 (22.4)	902 (21.3)	1522 (20.5)	
Rarely or never	6391 (41.8)	1370 (40.5)	1779 (42.0)	3141 (42.3)	
**Tea**					<0.001
Almost every day	3123 (20.8)	721 (21.3)	873 (20.6)	1530 (20.6)	
≥1 time/week	759 (5.0)	115 (3.4)	220 (5.2)	423 (5.7)	
≥1 time/mo	263 (1.7)	64 (1.9)	72 (1.7)	126 (1.7)	
Sometimes	472 (3.1)	132 (3.9)	140 (3.3)	201 (2.7)	
Rarely or never	10 428 (69.3)	2351 (69.5)	2931 (69.2)	5146 (69.3)	
**Medication**	8945 (59.5)	139 (4.1)	2546 (60.1)	6260 (84.3)	<0.001
**Nutritional supplement usage**	1728 (11.5)	281 (8.3)	415 (9.8)	1032 (13.9)	<0.001
**Cognitive impairment**	333 (2.2)	41 (1.2)	55 (1.3)	238 (3.2)	<0.001
**Public old age insurance**	8649 (57.5)	1854 (54.8)	2258 (53.3)	4537 (61.1)	<0.001
**Education level**					0.523†
Illiterate	3934 (26.1)	839 (24.8)	1038 (24.5)	2057 (27.7)	
Primary education	7267 (48.3)	1756 (51.9)	2207 (52.1)	3305 (44.5)	
Secondary education and above	3844 (25.6)	788 (23.3)	991 (23.4)	2064 (27.8)	
**Residential area**					<0.001
City	3149 (20.9)	480 (14.2)	716 (16.9)	1953 (26.3)	
Town	4462 (29.7)	1022 (30.2)	1309 (30.9)	2131 (28.7)	
Rural	7434 (49.4)	1881 (55.6)	2211 (52.2)	3342 (45.0)	

BMI – body mass index, SD – standard deviation, WHR – waist-to-hip ratio, x̄ – mean

*Pearson χ2 trend test, except.

†The Wilcoxon Rank Sum Test.

### Prevalence of multimorbidity and multimorbidity clusters

**Figure 2:**
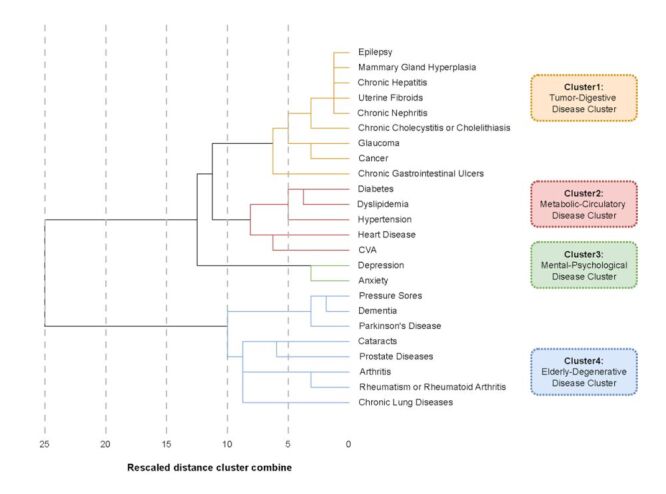
Dendrogram of cluster analysis showing three clusters.

The prevalent multimorbidity combinations were listed in **Table 2**. For the elderly with two chronic conditions in China, the most prevalent multimorbidity combination was hypertension and depression (2.1%), followed by depression and anxiety (2.0%), and hypertension and heart disease (1.9%). Among people with three chronic conditions, the most prevalent combination was hypertension, depression, and anxiety (1.0%). The prevalence of multimorbidity varied considerably between provinces, with the highest prevalence in Shanghai (575 / 646, 89.0%), followed by Heilongjiang (119 / 172, 69.2%) and Beijing (361/532, 67.9%) (Appendix S3 in the **Online Supplementary Document**). We identified four multimorbidity clusters among these individuals: the tumour-digestive disease cluster, the metabolic-circulatory disease cluster, the mental-psychological disease cluster, and the age-related degenerative disease cluster (**Figure 2**). The characteristics of participants in these four disease clusters were displayed in Appendices S4–5 in the **Online Supplementary Document**.

**Table 2:** Top 10 most prevalent combination of two and three chronic conditions among older adults in China

Variables	Sequence	Disease combination patterns	Total (n = 15 045)	Prevalence (%)
**Binary disease combinations**	1	Hypertension + depression	312	2.07
	2	Depression + anxiety	303	2.01
	3	Hypertension + heart disease	283	1.88
	4	Hypertension + diabetes	246	1.64
	5	Hypertension + CVA	215	1.43
	6	Hypertension + arthritis	130	0.86
	7	Hypertension + cataracts	124	0.82
	8	Hypertension + chronic lung diseases	100	0.66
	9	Chronic lung diseases + depression	71	0.47
	10	Heart disease + depression	61	0.41
**Ternary disease combinations**	1	Hypertension + depression + anxiety	145	0.96
	2	Hypertension + diabetes + heart disease	107	0.71
	3	Hypertension + heart disease + CVA	66	0.44
	4	Hypertension + diabetes + depression	55	0.37
	5	Hypertension + heart disease + depression	44	0.29
	6	Hypertension + CVA + depression	42	0.28
	7	Chronic lung diseases + depression + anxiety	29	0.19
	8	Heart disease + depression + anxiety	28	0.19
	9	Hypertension + heart disease + cataracts	23	0.15
	10	Hypertension + cataracts + depression	18	0.12

CVA – stroke and cerebrovascular diseases

The association rules extracted from the clusters were shown in Appendix S6 in the **Online Supplementary Document**. The tumour-digestive disease cluster contained nine relatively less prevalent conditions: epilepsy, mammary gland hyperplasia, chronic hepatitis, uterine fibroids, chronic nephritis, chronic cholecystitis or cholelithiasis, glaucoma, cancer, and chronic gastrointestinal ulcers. The ARM analysis revealed seven association rules, with chronic gastrointestinal ulcers and chronic cholecystitis or cholelithiasis being the most common consequents, each appearing in three rules.

The metabolic-circulatory disease cluster consisted of five conditions, including cardiovascular and metabolic diseases. The ARM in this cluster resulted in 13 association rules. Hypertension was found to be at the center of this cluster, appearing in 10 of the 13 association rules and having direct and indirect associations with almost all conditions in the cluster. The most common multimorbidity combination was heart disease and hypertension, with a support of 16.2%. The strongest association was found between dyslipidemia, stroke/cerebrovascular diseases, and heart disease (lift 3.7).

The mental-psychological disease cluster only contained two conditions: depression and anxiety. These conditions were highly associated, with a lift of 2.3 and a confidence of 72.9%, indicating a 73% probability of having depression when suffering from anxiety.

The age-related degenerative disease cluster comprised eight chronic diseases and generated a total of ten rules. Among these, four rules had cataracts as the consequent disease. Elderly individuals with rheumatism or rheumatoid arthritis and cataracts showed the highest probability of also having arthritis, with a confidence of 54.1%.

Cluster analysis by sex and region revealed comparable numbers and types of conditions across clusters, with some regional variations. In Northern China, five disease clusters were identified; however, the fifth cluster (comprising only hypertension and decubitus ulcers) did not yield any associated rules. The Eastern and Southern regions showed four similar disease clusters. The Central region highest number of association rules (n = 32), with the most frequent combination being diabetes, stroke, cerebrovascular diseases, and hypertension (Appendix S7 in the **Online Supplementary Document**). ARM analysis by sex groups showed similar multimorbidity combinations (Appendix S8 in the **Online Supplementary Document**).

### Association of influencing factors with multimorbidity clusters

The logistic regression analysis showed that age, gender, cognitive impairment, smoking, plant-based food, animal-based food, physical exercise, number of medications, marital status, residential area and education level were associated with multimorbidity among elderly individuals in China (**Table 3**).

**Table 3:** Top 10 most prevalent combination of two and three chronic conditions among older adults in China

Variables*	All		Tumour-digestive disease cluster		Metabolic-circulatory disease cluster		Mental-psychological disease cluster		Elderly-degenerative disease cluster	
	**β**	**OR (95% CI)**	**β**	**OR (95%CI)**	**β**	**OR (95%CI)**	**β**	**OR (95%CI)**	**β**	**OR (95%CI)**
Age	0.02	1.02 (1.01, 1.03)†	0.09	1.10 (0.92, 1.30)	0.02	1.02 (1.00, 1.05)	−0.03	0.97 (0.95, 0.99)‡	−0.02	0.99 (0.95, 1.02)
Gender (ref. female)	−0.52	0.59 (0.51, 0.70)†	−0.81	0.45 (0.03, 6.61)	−0.15	0.87 (0.59, 1.27)	−0.84	0.43 (0.31, 0.61)†	−0.75	0.47 (0.25, 0.91)§
BMI	0.01	1.00 (0.99, 1.01)	−0.24	0.78 (0.57, 1.08)	0.05	1.05 (1.02, 1.08)†	0.01	1.01 (1.00, 1.03)	−0.07	0.93 (0.87, 1.00)
WHR	−0.71	0.49 (0.23, 1.06)	−3.44	0.03 (9.283E¶-7, 1100.29)	−1.04	0.36 (0.06, 2.23)	−0.84	0.43 (0.31, 0.61)†	−2.87	0.06 (0.01, 1.48)
Cognitive impairment (ref. no)	1.24	3.46 (2.26, 5.30)†	−15.46	1.927E-7ǁ	−0.90	0.41 (0.07, 2.46)	1.73	5.66 (3.03, 10.57)†	−0.37	0.69 (0.11, 4.50)
Smoking status (ref. no)	0.36	1.44 (1.22, 1.69)†	−1.70	0.18 (0.01, 3.65)	0.05	1.05 (0.71, 1.55)	−0.01	0.99 (0.69, 1.42)	−0.35	0.70 (0.34, 1.45)
Drinking status (ref. no)	−0.01	1.00 (0.86, 1.16)	0.82	2.26 (0.27, 18.88)	0.09	1.10 (0.76, 1.58)	0.39	1.48 (1.08, 2.02)	0.05	1.05 (0.58, 1.92)
Physical exercise (ref. no)	−0.34	0.71 (0.58, 0.87)†	5.02	151.56 (2.467E-13, 9.310E+16)	−0.48	0.62 (0.38, 1.00)	−0.67	0.51 (0.36, 0.74)†	−0.25	0.78 (0.35, 1.72)
Plant-based food score	−0.05	0.95 (0.94, 0.97)†	0.21	1.23 (0.90, 1.68)	0.11	1.11 (1.07, 1.15)†	−0.09	0.92 (0.89, 0.94)†	0.03	1.03 (0.97, 1.09)
Animal-based food score	−0.03	0.97 (0.96, 0.99)†	0.32	1.37 (0.92, 2.05)	−0.08	0.92 (0.87, 0.98)‡	−0.03	0.97 (0.93, 1.01)	0.13	1.14 (1.05, 1.23)†
Highly processed foods score	0.01	1.01 (0.99, 1.04)	0.08	1.08 (0.83, 1.40)	−0.11	0.89 (0.85, 0.93)†	0.08	1.09 (1.03, 1.15)‡	0.02	1.22 (1.10, 1.36)†
No. medications	4.50	90.42 (75.37, 108.47)†	6.98	1071.27 (112.38, 10 211.83)†	7.82	2497.95 (1825.45, 3418.21)†	0.76	2.14 (1.42, 3.22)†	3.75	42.44 (28.98, 62.16)†
Nutritional supplement usage (ref. no)	0.17	1.19 (0.97, 1.45)	−18.72	7.440E-9	0.36	1.43 (0.91, 2.25)	−0.53	0.59 (0.35, 1.00)	0.59	1.81 (0.97, 3.36)
Residential status (ref. alone)	−0.08	0.92 (0.76, 1.12)	1.17	3.24 (0.08, 129.48)	−0.04	0.96 (0.60, 1.53)	−0.43	0.65 (0.46, 0.93)§	0.25	1.28 (0.59, 2.81)
Marital status (ref. never married)										
*Currently married*	−1.35	0.26 (0.15, 0.45)†	12.02	166 440.00 (14 963.69, 1851 299.97)†	0.50	1.65 (0.23, 11.94)†	−0.14	0.87 (0.31, 2.43)	−1.95	0.14 (0.02, 1.11)
*Divorced*	−1.23	0.29 (0.17, 0.50)†	12.29	216 345.97	0.34	1.40 (0.19, 10.17)†	0.01	1.01 (0.36, 2.82)	−1.31	0.27 (0.04, 2.08)
Public old age insurance (ref. no)	−0.09	0.92 (0.81, 1.04)	0.82	2.27 (0.26, 19.61)	−0.25	0.78 (0.58, 1.06)	−0.09	0.91 (0.71, 1.18)	−0.36	0.70 (0.42, 1.17)
Education (ref. illiterate)										
*Secondary education and above*	−0.12	0.89 (0.73, 1.08)	−0.25	0.78 (0.01, 46.53)	0.46	1.59 (0.99, 2.56)	−0.32	0.73 (0.48, 1.11)	0.60	1.83 (0.84, 3.98)
*Primary education*	−0.21	0.81 (0.70, 0.94)†	−0.76	0.47 (0.04, 6.19)	0.35	1.42 (0.98, 2.06)	−0.28	0.75 (0.57, 0.99)§	0.44	1.55 (0.87, 2.78)
Residential area (ref. rural)										
*City*	0.11	1.12 (0.92, 1.36)	−1.42	0.24 (0.01, 6.48)	−0.26	0.77 (0.51, 1.17)	−0.25	0.78 (0.48, 1.27)	−0.53	0.59 (0.28, 1.23)
*Town*	0.16	1.17 (1.03, 1.33)†	−0.80	0.45 (0.05, 4.34)	−0.10	0.90 (0.65, 1.25)	0.03	1.03 (0.80, 1.34)	−0.52	0.60 (0.34, 1.04)

BMI – body mass index, CI – confidence interval, OR – odds ratio, WHR – waist-to-hip ratio

*The healthy group is the reference group.

†*P* < 0.001

‡*P* < 0.01

§*P* < 0.05;

¶‘E’ stands for ‘exponent’ or ‘times 10 to the power of’.

ǁWhen a value is extremely small or large, SPSS defaults to using scientific notation and does not provide a confidence interval.

In the tumour-digestive disease cluster, the number of medications and marital status (currently married) were identified as risk factors. In the metabolic-circulatory disease cluster, body mass index (BMI), plant-based food score, number of medications, and marital status (currently married and divorced) were risk factors, while animal-based food score and highly processed food score were protective factors. In the mental-psychological disease cluster, cognitive impairment, highly processed food score, and number of medications were risk factors, whereas age, gender (male), waist-to-hip ratio (WHR), physical exercise, plant-based food score, living with others, and primary education level were protective factors. In age-related degenerative disease cluster, the animal-based food score, highly processed food score, and number of medications were risk factors, while gender (male) was a protective factor.

### Multimorbidity patterns

Four multimorbidity patterns were identified based on multimorbidity clusters and factors, including the tumour-digestive disease cluster (epilepsy, glaucoma, chronic nephritis, uterine fibroids, chronic cholecystitis or cholelithiasis, chronic gastrointestinal ulcers, cancer, chronic hepatitis, mammary gland hyperplasia, married, number of medication types), the metabolic-circulatory disease cluster (diabetes, dyslipidaemia, hypertension, stroke and cerebrovascular diseases, heart disease, BMI, plant-based foods, animal-based foods, highly processed foods, number of medication types, married or ever married), the mental-psychological disease cluster (anxiety, depression, age, male, WHR, cognitive impairment, physical activity or manual labour, plant-based foods, highly processed foods, number of medication types, living with others, primary school education), and the elderly-degenerative disease cluster (pressure sores, dementia, Parkinson disease, cataracts, prostate disease, arthritis, rheumatism or rheumatoid arthritis, chronic lung disease, male, animal-based foods, highly processed foods, number of medication types).

**Figure 3:**
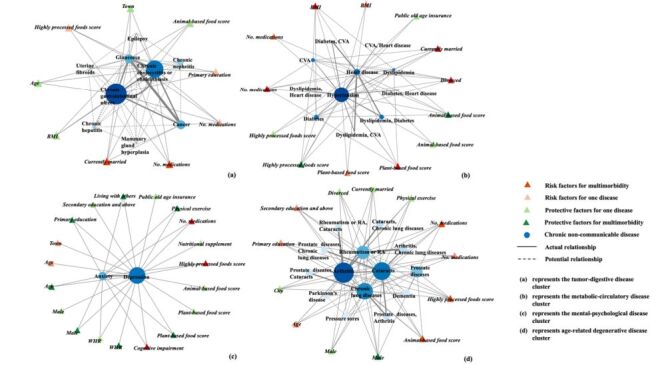
Network graphs for multimorbidity patterns. **Panel A.** Tumour-digestive disease cluster showing relationships between chronic conditions (blue nodes), risk factors (red triangles), and protective factors (green triangles). Key nodes include cancer, chronic gastrointestinal ulcers, and chronic hepatitis, with connections to lifestyle factors such as BMI and dietary patterns. **Panel B.** Metabolic-circulatory disease cluster illustrating the network of cardiovascular and metabolic conditions, centered around hypertension with strong connections to diabetes, stroke and cerebrovascular diseases, and heart disease. **Panel C.** Mental-psychological disease cluster depicting depression as a central node, connected to anxiety and cognitive impairment, along with various demographic and lifestyle factors. **Panel D.** Age-related degenerative disease cluster showing the interconnections between arthritis, cataracts, and chronic lung diseases, with multiple connections to lifestyle and demographic factors. Solid lines represent actual relationships while dashed lines indicate potential relationships. Node colors indicate chronic non-communicable diseases (blue circles), risk factors for multimorbidity (dark red triangles), risk factors for one disease (light red triangles), protective factors for one disease (light green triangles), and protective factors for multimorbidity (dark green triangles).

The first network had nine disease nodes and nine factor nodes, with gastrointestinal ulcer being the largest disease node. Marital status (currently married) and number of medications were the risk factors in this network (**Figure 3**, Panel A). The second network consisted of eleven disease nodes and thirteen factor nodes, with hypertension being the predominant disease node. The number of medications, plant-based food score, marital status (currently married and divorced) and BMI were risk factors, while the animal-based food score and the highly processed food score acted as protective factors (**Figure 3**, Panel B). The third network had two disease nodes, depression and anxiety, and nineteen factor nodes (**Figure 3**, Panel C). The fourth network included fourteen disease nodes and thirteen factor nodes. Male was identified as a protective factor, whereas animal-based food score, highly processed food score and number of medications were risk factors (**Figure 3**, Panel D).

## DISCUSSION

This study examined the prevalence, patterns, and influencing factors of multimorbidity among older adults using data from CLHLS. Our finding revealed that 49.4% of older population experienced multimorbidity, a prevalence rate consistent with previous studies conducted in China [18] but higher than those reported in some developed countries [44]. The most common binary disease combination was hypertension and depression, while the most frequent ternary combination was hypertension, depression and anxiety. Four major multimorbidity clusters were identified: the tumour-digestive disease cluster, the metabolic-circulatory disease cluster, the mental-psychological disease cluster, and the age-related degenerative disease cluster. Gastrointestinal ulcers, cholecystitis or cholelithiasis, hypertension, depression, and cataracts were the most frequently occurring conditions within these four clusters.

The formation of multimorbidity clusters likely reflects the high frequency and co-occurrence of specific diseases, as identified through association rule analysis. These clusters may also share common pathophysiological mechanisms and biological pathways. For example, the co-occurrence of hypertension with metabolic and cardiovascular diseases may be linked to endothelial dysfunction and metabolic syndrome [45,46]. Similarly, mental health conditions often cluster due to shared factors like inflammation, oxidative stress, and neuroendocrine disruptions [47–49]. In age-related degenerative diseases, mitochondrial dysfunction and impaired tissue repair are likely contributors [50,51].

Geographic region significantly influenced multimorbidity patterns, highlighting the role of environmental and economic factors in chronic disease relationships [17,23]. Additionally, gender and medication use consistently impacted all disease clusters. Other factors, including BMI, WHR, cognitive impairment, dietary patterns (plant-based, animal-based, and highly processed foods), and marital status, showed varying effects across different clusters.

In terms of personal characteristics, a higher BMI was associated with increased risk of multimorbidity in the metabolic-circulatory disease cluster, likely due to chronic inflammation and insulin resistance caused by excess adipose tissue. These processes disrupt glucose and lipid metabolism, leading to endothelial dysfunction, atherosclerosis, and elevated cardiovascular and metabolic disease risks through altered adipokine profiles and oxidative stress [52–56]. In contrast, WHR was a protective factor in the mental-psychological disease cluster, potentially linked to severe appetite loss and weight reduction often seen in mental disorders [57].

For lifestyle characteristics, while plant-based diets are widely recognised for their cardioprotective effects [58,59], our findings identified plant-based foods as a risk factor for multimorbidity in the metabolic-circulatory disease cluster. This discrepancy may result from dietary changes adopted after diagnosis, driven by medical advice or self-initiated interventions, potentially introducing bias in assessing their true effects. Plant-based diets, such as the Mediterranean and DASH diets, are known to modulate gut microbiota, promoting protective microbes that produce short-chain fatty acids with immunomodulatory, antihypertensive, and cardioprotective properties [60]. Conversely, we observed a protective effect of plant-based foods in the mental-psychological disease cluster, consistent with studies linking diets rich in fruits, vegetables, and nuts to improved mental health outcomes. These effects are attributed to reduced systemic inflammation and enhanced gut microbiota diversity, which play roles in supporting mental well-being [61].

Animal-based foods showed a protective effect in the metabolic-circulatory disease cluster but were a risk factor in the age-related degenerative disease cluster, reflecting their complex role in health outcomes. In the metabolic-circulatory cluster, their protective effect may stem from high-quality protein, essential amino acids, and micronutrients like iron and vitamin B12, which support metabolic and cardiovascular health [62,63]. Conversely, in the age-related degenerative cluster, animal-based foods may elevate the production of prostaglandins and leukotrienes, promoting cellular aging, tissue degeneration, and the progression of degenerative diseases [64,65].

Highly processed foods were identified as a protective factor in the metabolic-circulatory disease cluster but a risk factor in the mental-psychological and age-related degenerative clusters. These foods, often high in sugars, salts, unhealthy fats, and additives, may lead to nutritional imbalances and deficiencies, accelerating cognitive and physical decline in aging populations and increasing multimorbidity risk [66–68]. In the metabolic-circulatory cluster, the protective effect could reflect healthier dietary adjustments by diabetic patients, such as reduced sugar intake and weight control, compared to less restrictive habits in other populations [69]. However, the cross-sectional design limits causal inferences and the determination of directionality in these associations. Additionally, regular physical activity emerged as a protective factor in the mental-psychological cluster, likely due to its physical health benefits and its ability to release mood-enhancing endorphins [70–72].

In the socioeconomic characteristics, marital status (currently married or divorced) was associated with a higher risk of multimorbidity in the metabolic-circulatory disease cluster [52]. This may stem from marital conflicts, which trigger stress responses and elevate cortisol levels [73]. Chronic cortisol elevation disrupts glucose regulation, raises blood pressure, and alters lipid metabolism-key risk factors for cardiovascular disease [74]. Prolonged stress can accelerate cardiovascular aging and increase the risk of heart disease and stroke [75,76]. Additionally, marital stress may lead to harmful coping behaviours, such as smoking, further exacerbating cardiovascular risks. Conversely, living with others and attaining at least a primary education were protective factors in the mental-psychological disease cluster. Co-habitation provides emotional support and reduces loneliness, key contributors to mental health issues [77,78]. Basic education enhances health literacy and communication skills, enabling better access to health services and healthier stress management strategies [79,80].

Gender was a protective factor for multimorbidity across all disease clusters, particularly within the mental-psychological cluster. In East Asian cultures, men are often socialised to be resilient, which may promote proactive stress management strategies [81,82]. Conversely, polypharmacy was consistently associated with higher risks of multimorbidity across all clusters, reflecting the increased burden of chronic disease [83]. Polypharmacy heightens the risk of drug-drug interactions and side effects, potentially harming patient health [84]. Effective medication management is essential for optimising treatment outcomes and improving quality of life. Healthcare providers should prioritise rational prescribing, minimise unnecessary medications, and conduct comprehensive assessments to reduce adverse drug events and enhance therapeutic efficacy [85,86].

Based on our analysis of the common characteristics and unique features of different multimorbidity clusters, we propose targeted intervention strategies to guide health care providers and systems in delivering more personalised patient care. For the metabolic-circulatory disease cluster, a comprehensive metabolic risk assessment and dietary counselling focused on nutrient balance are essential. In the mental-psychological disease cluster, interventions should prioritise holistic care, including integrated mental health screenings, physical activity programmes promoting both physical and mental well-being, and support systems to address social isolation, especially for older individuals living alone. In the age-related degenerative disease cluster, preventive strategies should include nutritional counselling to limit highly processed foods and regular cognitive function assessments. For all clusters, medication review protocols are crucial to minimise polypharmacy risks. Additionally, health care systems should develop multidisciplinary care teams and digital health tools for personalised risk tracking and intervention.

Cardiovascular-metabolic disease was the most common multimorbidity cluster identified in published studies [29,87], and a systematic review of the disease pattern among the elderly in high-income countries also confirmed the prevalence of this cluster [88]. However, unlike other studies that reported a relationship between hypertension and arthritis [18,23], our analysis did not confirm this correlation. This discrepancy may be due to our study combined approach of clustering and ARM, which effectively reduced the statistical bias associated high incidence rates due to random co-occurrence. Our study also identified a multimorbidity pattern similar to that observed in a national study which enrolled 9072 individuals aged 60 and above, including various diseases and their influencing factors [26]. In this study, observed-to-expected (O/E) ratios were used to investigate associations between conditions, and χ^2^ tests and logistic regression were used to explore relationships between 13 conditions and their influencing factors. Then they adopted the non-overlapping cluster detection algorithm and identified five multimorbidity patterns. The factors we have included were referenced from the ‘Petal’ theoretical framework in this study; however, we had considered further lifestyle behavioural dimensions such as nutritional and dietary habits. The multimorbidity patterns we identified show similarities to those in this study, particularly in terms of the coexistence of cardiovascular-metabolic diseases associated with high BMI, as well as the clustering of digestive, kidney, liver diseases, and cancer.

### Strength and limitations

Recent research has increasingly utilised advanced analytical methods to explore correlations among chronic disease multimorbidities. However, latent categorical variable analysis (LCA) provides only limited information about disease associations [24], while cluster analyses fail to capture the strength of the associations. Moreover, relying solely on association rule mining (ARM) can generate an overwhelming number of frequent items sets and rules, making it challenging to interpret the results. To address these limitations, we enhanced previous approaches by integrating ARM with cluster analysis. This combined method effectively reduced the data volume for ARM, allowing for more precise identification of association rules between chronic diseases and multimorbidity clusters. Each cluster was analysed with tailored minimum confidence levels based on varying disease frequencies. Additionally, we examined the characteristic correlations within specific multimorbidity clusters to offer insights for developing targeted health care strategies that address the unique needs of patients with multiple chronic conditions. Furthermore, we included individual and social factors that were statistically significant in prior studies, with an emphasis on the impact of lifestyle behaviours on specific multimorbidity clusters. Finally, we presented the results using network graphs to enhance the clarity and comprehension of our findings.

However, our study has several limitations. First, the cross-sectional design may have introduced recall and reporting biases, and it limited our ability to establish causality between the disease and associated factors. Second, although our study included 22 chronic disease items, variations in how diseases were classified in the questionnaire often led to several conditions being grouped under the same category. This grouping could affect the accuracy of the multimorbidity combinations we explored. Third, the questionnaire survey lacked detailed information on the duration and severity of chronic conditions, as well as the duration of nutritional supplement use. The absence of such detailed data may introduce confounding factors and limit a more in-depth analysis of the relationships between these factors and multimorbidity.

## CONCLUSIONS

Multimorbidity is highly prevalent among the elderly population, with hypertension and depression being the most common co-occurring conditions. Four distinct multimorbidity patterns were identified, and the influencing factors varies across these clusters. This highlights the need for tailored management strategies specific to each multimorbidity cluster rather than a one-size-fits-all approach. Future prospective cohort studies are needed to further investigate the factors influencing multimorbidity and to establish causal relationships.

## Additional material


Online Supplementary Document

## References

[1] Zhao YW Haregu TN He L Lu S Katar A Wang H The effect of multimorbidity on functional limitations and depression amongst middle-aged and older population in China: a nationwide longitudinal study. Age Ageing. 2021;50:190–7. 10.1093/ageing/afaa117 32556149

[2] National Bureau of Statistics Seventh National Census Bulletin(No. 5). Available: http://www.stats.gov.cn/tjsj/tjgb/rkpcgb/qgrkpcgb/202106/t20210628_1818824.html. Accessed: 11 May 2021.

[3] Smith SM O’Dowd T Chronic diseases: what happens when they come in multiples? Br J Gen Pract. 2007;57:268–70. 17394728 PMC2043326

[4] Barnett K Mercer SW Norbury M Watt G Wyke S Guthrie B Epidemiology of multimorbidity and implications for health care, research, and medical education: a cross-sectional study. Lancet. 2012;380:37–43. 10.1016/S0140-6736(12)60240-2 22579043

[5] van Oostrom SH Gijsen R Stirbu I Korevaar JC Schellevis FG Picavet HS Time Trends in Prevalence of Chronic Diseases and Multimorbidity Not Only due to Aging: Data from General Practices and Health Surveys. PLoS One. 2016;11:e0160264. 10.1371/journal.pone.0160264 27482903 PMC4970764

[6] van den Akker M Buntinx F Metsemakers JF Roos S Knottnerus JA Multimorbidity in general practice: prevalence, incidence, and determinants of co-occurring chronic and recurrent diseases. J Clin Epidemiol. 1998;51:367–75. 10.1016/S0895-4356(97)00306-5 9619963

[7] Kingston A Robinson L Booth H Knapp M Jagger C Projections of multi-morbidity in the older population in England to 2035: estimates from the Population Ageing and Care Simulation (PACSim) model. Age Ageing. 2018;47:374–80. 10.1093/ageing/afx201 29370339 PMC5920286

[8] Kadam UT Croft PR Clinical multimorbidity and physical function in older adults: a record and health status linkage study in general practice. Fam Pract. 2007;24:412–9. 10.1093/fampra/cmm049 17698977

[9] Gijsen R Hoeymans N Schellevis FG Ruwaard D Satariano WA van den Bos GA Causes and consequences of comorbidity: a review. J Clin Epidemiol. 2001;54:661–74. 10.1016/S0895-4356(00)00363-2 11438406

[10] Fortin M Lapointe L Hudon C Vanasse A Ntetu AL Maltais D Multimorbidity and quality of life in primary care: a systematic review. Health Qual Life Outcomes. 2004;2:51. 10.1186/1477-7525-2-51 15380021 PMC526383

[11] Yang G Kong L Zhao W Wan X Zhai Y Chen LC Emergence of chronic non-communicable diseases in China. Lancet. 2008;372:1697–705. 10.1016/S0140-6736(08)61366-5 18930526

[12] Pearson-Stuttard J Ezzati M Gregg EW Multimorbidity a defining challenge for health systems. Lancet Public Health. 2019;4:e599–600. 10.1016/S2468-2667(19)30222-1 31812234

[13] Boyd CM Kent DM Evidence-based medicine and the hard problem of multimorbidity. J Gen Intern Med. 2014;29:552–3. 10.1007/s11606-013-2658-z 24442331 PMC3965745

[14] Boyd CM Darer J Boult C Fried LP Boult L Wu AW Clinical practice guidelines and quality of care for older patients with multiple comorbid diseases: implications for pay for performance. JAMA. 2005;294:716–24. 10.1001/jama.294.6.716 16091574

[15] Guthrie B Payne K Alderson P McMurdo ME Mercer SW Adapting clinical guidelines to take account of multimorbidity. BMJ. 2012;345:e6341. 10.1136/bmj.e6341 23036829

[16] Gu J Chao J Chen W Xu H Zhang R He T Multimorbidity and health-related quality of life among the community-dwelling elderly: A longitudinal study. Arch Gerontol Geriatr. 2018;74:133–40. 10.1016/j.archger.2017.10.019 29096228

[17] Chen S Wang S Jia W Han K Song Y Liu S Spatiotemporal Analysis of the Prevalence and Pattern of Multimorbidity in Older Chinese Adults. Front Med (Lausanne). 2022;8:806616. 10.3389/fmed.2021.806616 35127761 PMC8811186

[18] Han S Mo G Gao T Sun Q Liu H Zhang M Age, sex, residence, and region-specific differences in prevalence and patterns of multimorbidity among older Chinese: evidence from Chinese Longitudinal Healthy Longevity Survey. BMC Public Health. 2022;22:1116. 10.1186/s12889-022-13506-0 35658851 PMC9166487

[19] Liu C Shu R Liang H Liang Y Multimorbidity Patterns and the Disablement Process among Public Long-Term Care Insurance Claimants in the City of Yiwu (Zhejiang Province, China). Int J Environ Res Public Health. 2022;19:645. 10.3390/ijerph19020645 35055466 PMC8775810

[20] Hsieh PI Chen YC Chen TF Chiou JM Chen JH Multimorbid Patterns and Cognitive Performance in the Presence of Informative Dropout Among Community-Dwelling Taiwanese Older Adults. Innov Aging. 2023;7:igad012. 10.1093/geroni/igad012 37007640 PMC10053640

[21] Zhong Y Qin G Xi H Cai D Wang Y Wang T Prevalence, patterns of multimorbidity and associations with health care utilization among middle-aged and older people in China. BMC Public Health. 2023;23:537. 10.1186/s12889-023-15412-5 36944960 PMC10031889

[22] Shi Z Zhang Z Shi K Yu B Jiang Z Yang L Association between multimorbidity trajectories and incident disability among mid to older age adults: China Health and Retirement Longitudinal Study. BMC Geriatr. 2022;22:741. 10.1186/s12877-022-03421-9 36096760 PMC9469590

[23] Chen Y Shi L Zheng X Yang J Xue Y Xiao S Patterns and Determinants of Multimorbidity in Older Adults: Study in Health-Ecological Perspective. Int J Environ Res Public Health. 2022;19:16756. 10.3390/ijerph192416756 36554647 PMC9779369

[24] Zhang Q Han X Zhao X Wang Y Multimorbidity patterns and associated factors in older Chinese: results from the China health and retirement longitudinal study. BMC Geriatr. 2022;22:470. 10.1186/s12877-022-03154-9 35641904 PMC9158229

[25] Lu J Wang Y Hou L Zuo Z Zhang N Wei A Multimorbidity patterns in old adults and their associated multi-layered factors: a cross-sectional study. BMC Geriatr. 2021;21:372. 10.1186/s12877-021-02292-w 34147073 PMC8214251

[26] Xiaomin M. Multimorbidity Pattern Mining and Prevention and Control Mode of Chronic Diseases in the Elderly. 2021.

[27] Zhang C Xiao S Shi L Xue Y Zheng X Dong F Urban-Rural Differences in Patterns and Associated Factors of Multimorbidity Among Older Adults in China: A Cross-Sectional Study Based on Apriori Algorithm and Multinomial Logistic Regression. Front Public Health. 2021;9:707062. 10.3389/fpubh.2021.707062 34527650 PMC8437131

[28] Méndez-Flores JJ Marroquín-Cosar RE Bernabé-Ortiz A Multimorbidity and Sleep Patterns among Adults in a Peruvian Semi-Urban Area. Sleep Sci. 2023;16:51–8. 10.1055/s-0043-1767755 37151763 PMC10157817

[29] Lin WQ Yuan LX Sun MY Wang C Liang EM Li YH Prevalence and patterns of multimorbidity in chronic diseases in Guangzhou, China: a data mining study in the residents’ health records system among 31 708 community-dwelling elderly people. BMJ Open. 2022;12:e056135. 10.1136/bmjopen-2021-056135 35613781 PMC9134174

[30] Ronaldson A Arias de la Torre J Prina M Armstrong D Das-Munshi J Hatch S Associations between physical multimorbidity patterns and common mental health disorders in middle-aged adults: A prospective analysis using data from the UK Biobank. Lancet Reg Health Eur. 2021;8:100149. 10.1016/j.lanepe.2021.100149 34557851 PMC8447568

[31] Roomaney RA van Wyk B Cois A Pillay van-Wyk V Multimorbidity patterns in South Africa: A latent class analysis. Front Public Health. 2023;10:1082587. 10.3389/fpubh.2022.1082587 36711391 PMC9875075

[32] Nichols L Taverner T Crowe F Richardson S Yau C Kiddle S In simulated data and health records, latent class analysis was the optimum multimorbidity clustering algorithm. J Clin Epidemiol. 2022;152:164–75. 10.1016/j.jclinepi.2022.10.011 36228971 PMC7613854

[33] Zeng Y Feng Q Hesketh T Christensen K Vaupel JW Survival, disabilities in activities of daily living, and physical and cognitive functioning among the oldest-old in China: a cohort study. Lancet. 2017;389:1619–29. 10.1016/S0140-6736(17)30548-2 28285816 PMC5406246

[34] Lv X Li W Ma Y Chen H Zeng Y Yu X Cognitive decline and mortality among community-dwelling Chinese older people. BMC Med. 2019;17:63. 10.1186/s12916-019-1295-8 30871536 PMC6419492

[35] Wei K Liu Y Yang J Gu N Cao X Zhao X Living arrangement modifies the associations of loneliness with adverse health outcomes in older adults: evidence from the CLHLS. BMC Geriatr. 2022;22:59. 10.1186/s12877-021-02742-5 35038986 PMC8764854

[36] Qi R Sheng B Zhou L Chen Y Sun L Zhang X Association of Plant-Based Diet Indices and Abdominal Obesity with Mental Disorders among Older Chinese Adults. Nutrients. 2023;15:2721. 10.3390/nu15122721 37375625 PMC10303527

[37] Zhu A Chen H Shen J Wang X Li Z Zhao A Interaction between plant-based dietary pattern and air pollution on cognitive function: a prospective cohort analysis of Chinese older adults. Lancet Reg Health West Pac. 2022;20:100372. 10.1016/j.lanwpc.2021.100372 35028630 PMC8741490

[38] Liang F Fu J Turner-McGrievy G Wang Y Qiu N Ding K Association of Body Mass Index and Plant-Based Diet with Cognitive Impairment among Older Chinese Adults: A Prospective, Nationwide Cohort Study. Nutrients. 2022;14:3132. 10.3390/nu14153132 35956314 PMC9370436

[39] Zeng Y, Jr DP, Vlosky DA, Gu D, editors. Healthy longevity in China: demographic, socioeconomic, and psychological dimensions. Dordrecht: Springer; 2008.

[40] Cummings P Missing data and multiple imputation. JAMA Pediatr. 2013;167:656–61. 10.1001/jamapediatrics.2013.1329 23699969

[41] Obulkasim A van de Wiel MA HCsnip: An R Package for Semi-supervised Snipping of the Hierarchical Clustering Tree. Cancer Inform. 2015;14:1–19. 10.4137/CIN.S22080 25861213 PMC4372030

[42] Mahmoodi SA Mirzaie K Mahmoudi SM A new algorithm to extract hidden rules of gastric cancer data based on ontology. Springerplus. 2016;5:312. 10.1186/s40064-016-1943-9 27066344 PMC4786510

[43] Peng M Sundararajan V Williamson T Minty EP Smith TC Doktorchik CTA Exploration of association rule mining for coding consistency and completeness assessment in inpatient administrative health data. J Biomed Inform. 2018;79:41–7. 10.1016/j.jbi.2018.02.001 29425732

[44] Zemedikun DT Gray LJ Khunti K Davies MJ Dhalwani NN Patterns of Multimorbidity in Middle-Aged and Older Adults: An Analysis of the UK Biobank Data. Mayo Clin Proc. 2018;93:857–66. 10.1016/j.mayocp.2018.02.012 29801777

[45] Ungvari Z Tarantini S Kiss T Wren JD Giles CB Griffin CT Endothelial dysfunction and angiogenesis impairment in the ageing vasculature. Nat Rev Cardiol. 2018;15:555–65. 10.1038/s41569-018-0030-z 29795441 PMC6612360

[46] Kim JA Montagnani M Koh KK Quon MJ Reciprocal relationships between insulin resistance and endothelial dysfunction: molecular and pathophysiological mechanisms. Circulation. 2006;113:1888–904. 10.1161/CIRCULATIONAHA.105.563213 16618833

[47] Miller AH Raison CL The role of inflammation in depression: from evolutionary imperative to modern treatment target. Nat Rev Immunol. 2016;16:22–34. 10.1038/nri.2015.5 26711676 PMC5542678

[48] Zuo C Cao H Song Y Gu Z Huang Y Yang Y Nrf2: An all-rounder in depression. Redox Biol. 2022;58:102522. 10.1016/j.redox.2022.102522 36335763 PMC9641011

[49] Brewerton TD Lydiard RB Laraia MT Shook JE Ballenger JC CSF beta-endorphin and dynorphin in bulimia nervosa. Am J Psychiatry. 1992;149:1086–90. 10.1176/ajp.149.8.1086 1353317

[50] Fearon U Canavan M Biniecka M Veale DJ Hypoxia, mitochondrial dysfunction and synovial invasiveness in rheumatoid arthritis. Nat Rev Rheumatol. 2016;12:385–97. 10.1038/nrrheum.2016.69 27225300

[51] Schapira AH Mitochondria in the aetiology and pathogenesis of Parkinson’s disease. Lancet Neurol. 2008;7:97–109. 10.1016/S1474-4422(07)70327-7 18093566

[52] Abolnezhadian F Hosseini SA Alipour M Zakerkish M Cheraghian B Ghandil P Association Metabolic Obesity Phenotypes with Cardiometabolic Index, Atherogenic Index of Plasma and Novel Anthropometric Indices: A Link of FTO-rs9939609 Polymorphism. Vasc Health Risk Manag. 2020;16:249–56. 10.2147/VHRM.S251927 32612360 PMC7322142

[53] Su L Wu S Fu J Sun S Effects of Physical Activity, VO(2max), and Visfatin on Relationship Between BMI and Chronic Inflammation. Diabetes Metab Syndr Obes. 2024;17:4489–500. 10.2147/DMSO.S473266 39619219 PMC11607996

[54] Morigny P Houssier M Mouisel E Langin D Adipocyte lipolysis and insulin resistance. Biochimie. 2016;125:259–66. 10.1016/j.biochi.2015.10.024 26542285

[55] Lu X Xie Q Pan X Zhang R Zhang X Peng G Type 2 diabetes mellitus in adults: pathogenesis, prevention and therapy. Signal Transduct Target Ther. 2024;9:262. 10.1038/s41392-024-01951-9 39353925 PMC11445387

[56] Niemann B Rohrbach S Miller MR Newby DE Fuster V Kovacic JC Oxidative Stress and Cardiovascular Risk: Obesity, Diabetes, Smoking, and Pollution: Part 3 of a 3-Part Series. J Am Coll Cardiol. 2017;70:230–51. 10.1016/j.jacc.2017.05.043 28683970 PMC5568826

[57] Kroemer NB Opel N Teckentrup V Li M Grotegerd D Meinert S Functional Connectivity of the Nucleus Accumbens and Changes in Appetite in Patients With Depression. JAMA Psychiatry. 2022;79:993–1003. 10.1001/jamapsychiatry.2022.2464 36001327 PMC9403857

[58] De Filippo C Cavalieri D Di Paola M Ramazzotti M Poullet JB Massart S Impact of diet in shaping gut microbiota revealed by a comparative study in children from Europe and rural Africa. Proc Natl Acad Sci U S A. 2010;107:14691–6. 10.1073/pnas.1005963107 20679230 PMC2930426

[59] David LA Maurice CF Carmody RN Gootenberg DB Button JE Wolfe BE Diet rapidly and reproducibly alters the human gut microbiome. Nature. 2014;505:559–63. 10.1038/nature12820 24336217 PMC3957428

[60] Jama HA Beale A Shihata WA Marques FZ The effect of diet on hypertensive pathology: is there a link via gut microbiota-driven immunometabolism? Cardiovasc Res. 2019;115:1435–47. 10.1093/cvr/cvz091 30951169

[61] Nabavi SF Habtemariam S Di Lorenzo A Sureda A Khanjani S Nabavi SM Post-Stroke Depression Modulation and in Vivo Antioxidant Activity of Gallic Acid and Its Synthetic Derivatives in a Murine Model System. Nutrients. 2016;8:248. 10.3390/nu8050248 27136579 PMC4882661

[62] Wolfe RR Baum JI Starck C Moughan PJ Factors contributing to the selection of dietary protein food sources. Clin Nutr. 2018;37:130–8. 10.1016/j.clnu.2017.11.017 29233589

[63] Martens JH Barg H Warren MJ Jahn D Microbial production of vitamin B12. Appl Microbiol Biotechnol. 2002;58:275–85. 10.1007/s00253-001-0902-7 11935176

[64] Rondanelli M Faliva MA Miccono A Naso M Nichetti M Riva A Food pyramid for subjects with chronic pain: foods and dietary constituents as anti-inflammatory and antioxidant agents. Nutr Res Rev. 2018;31:131–51. 10.1017/S0954422417000270 29679994

[65] Limketkai BN Iheozor-Ejiofor Z Gjuladin-Hellon T Parian A Matarese LE Bracewell K Dietary interventions for induction and maintenance of remission in inflammatory bowel disease. Cochrane Database Syst Rev. 2019;2:CD012839. 10.1002/14651858.CD012839.pub2 30736095 PMC6368443

[66] Rondanelli M Perdoni F Peroni G Caporali R Gasparri C Riva A Ideal food pyramid for patients with rheumatoid arthritis: A narrative review. Clin Nutr. 2021;40:661–89. 10.1016/j.clnu.2020.08.020 32928578

[67] Li H Li S Yang H Zhang Y Zhang S Ma Y Association of Ultraprocessed Food Consumption With Risk of Dementia: A Prospective Cohort Study. Neurology. 2022;99:e1056–66. 10.1212/WNL.0000000000200871 36219796

[68] Zhang H Greenwood DC Risch HA Bunce D Hardie LJ Cade JE Meat consumption and risk of incident dementia: cohort study of 493,888 UK Biobank participants. Am J Clin Nutr. 2021;114:175–84. 10.1093/ajcn/nqab028 33748832 PMC8246598

[69] Mozaffarian D Dietary and Policy Priorities for Cardiovascular Disease, Diabetes, and Obesity: A Comprehensive Review. Circulation. 2016;133:187–225. 10.1161/CIRCULATIONAHA.115.018585 26746178 PMC4814348

[70] Laird E Rasmussen CL Kenny RA Herring MP Physical Activity Dose and Depression in a Cohort of Older Adults in The Irish Longitudinal Study on Ageing. JAMA Netw Open. 2023;6:e2322489. 10.1001/jamanetworkopen.2023.22489 37428505 PMC10334250

[71] Chen PJ Chen KM Hsu HF Belcastro F Types of exercise and training duration on depressive symptoms among older adults in long-term care facilities. Ageing Res Rev. 2022;77:101613. 10.1016/j.arr.2022.101613 35339704

[72] Pratley RE Hagberg JM Dengel DR Rogus EM Muller DC Goldberg AP Aerobic exercise training-induced reductions in abdominal fat and glucose-stimulated insulin responses in middle-aged and older men. J Am Geriatr Soc. 2000;48:1055–61. 10.1111/j.1532-5415.2000.tb04780.x 10983904

[73] Baker B Paquette M Szalai JP Driver H Perger T Helmers K The influence of marital adjustment on 3-year left ventricular mass and ambulatory blood pressure in mild hypertension. Arch Intern Med. 2000;160:3453–8. 10.1001/archinte.160.22.3453 11112239

[74] Spruill TM Chronic psychosocial stress and hypertension. Curr Hypertens Rep. 2010;12:10–6. 10.1007/s11906-009-0084-8 20425153 PMC3694268

[75] Orth-Gomér K Wamala SP Horsten M Schenck-Gustafsson K Schneiderman N Mittleman MA Marital stress worsens prognosis in women with coronary heart disease: The Stockholm Female Coronary Risk Study. JAMA. 2000;284:3008–14. 10.1001/jama.284.23.3008 11122587

[76] Blom M Janszky I Balog P Orth-Gomér K Wamala SP Social relations in women with coronary heart disease: the effects of work and marital stress. J Cardiovasc Risk. 2003;10:201–6. 10.1097/01.hjr.0000065926.57001.e0 12775953

[77] Hou B Zhang H Latent profile analysis of depression among older adults living alone in China. J Affect Disord. 2023;325:378–85. 10.1016/j.jad.2022.12.154 36640808

[78] Hu C Dai Z Liu H Liu S Du M Liu T Decomposition and comparative analysis of depressive symptoms between older adults living alone and with others in China. Front Public Health. 2023;11:1265834. 10.3389/fpubh.2023.1265834 37809006 PMC10556662

[79] Mezuk B Eaton WW Golden SH Ding Y The influence of educational attainment on depression and risk of type 2 diabetes. Am J Public Health. 2008;98:1480–5. 10.2105/AJPH.2007.126441 18556604 PMC2446469

[80] Bracke P van de Straat V Missinne S Education, mental health, and education-labor market misfit. J Health Soc Behav. 2014;55:442–59. 10.1177/0022146514557332 25413804

[81] Seidler ZE Dawes AJ Rice SM Oliffe JL Dhillon HM The role of masculinity in men’s help-seeking for depression: A systematic review. Clin Psychol Rev. 2016;49:106–18. 10.1016/j.cpr.2016.09.002 27664823

[82] Parker G Fletcher K Paterson A Anderson J Hong M Gender differences in depression severity and symptoms across depressive sub-types. J Affect Disord. 2014;167:351–7. 10.1016/j.jad.2014.06.018 25020270

[83] Nicholson K Liu W Fitzpatrick D Hardacre KA Roberts S Salerno J Prevalence of multimorbidity and polypharmacy among adults and older adults: a systematic review. Lancet Healthy Longev. 2024;5:e287–96. 10.1016/S2666-7568(24)00007-2 38452787

[84] Calderón-Larrañaga A Vetrano DL Ferrucci L Mercer SW Marengoni A Onder G Multimorbidity and functional impairment-bidirectional interplay, synergistic effects and common pathways. J Intern Med. 2019;285:255–71. 10.1111/joim.12843 30357990 PMC6446236

[85] Reeve J Maden M Hill R Turk A Mahtani K Wong G Deprescribing medicines in older people living with multimorbidity and polypharmacy: the TAILOR evidence synthesis. Health Technol Assess. 2022;26:1–148. 10.3310/AAFO2475 35894932 PMC9376985

[86] Hasan Ibrahim AS Barry HE Hughes CM A systematic review of general practice-based pharmacists’ services to optimize medicines management in older people with multimorbidity and polypharmacy. Fam Pract. 2021;38:509–23. 10.1093/fampra/cmaa146 33506870

[87] Wang XX Lin WQ Chen XJ Lin YY Huang LL Zhang SC Multimorbidity associated with functional independence among community-dwelling older people: a cross-sectional study in Southern China. Health Qual Life Outcomes. 2017;15:73. 10.1186/s12955-017-0635-7 28412945 PMC5392938

[88] Prados-Torres A Calderón-Larrañaga A Hancco-Saavedra J Poblador-Plou B van den Akker M Multimorbidity patterns: a systematic review. J Clin Epidemiol. 2014;67:254–66. 10.1016/j.jclinepi.2013.09.021 24472295

