# Supplementary material for: Multimorbidity patterns and influencing factors in older Chinese adults: a national population-based cross-sectional survey
Source: J Glob Health. 2025 Feb 21;15:04051. doi: 10.7189/jogh.15.04051 (PMC11843521; doi:10.7189/jogh.15.04051)
Supplement: Online Supplementary Document [file jogh-15-04051-s001.pdf]

Appendix S1. Specific food items and scoring criteria

Food groups	Category	Frequency	Score
**Plant-based food**	Whole grain	Yes	5
		No	1
	Vegetable oil	Yes	5
		No	1
	Fruits	Almost every day	5
		Often	4
		Sometimes	2
		Rarely or never	1
	Vegetables	Almost every day	5
		Often	4
		Sometimes	2
		Rarely or never	1
	Garlic	almost every day	5
		≥1 time/week	4
		≥1 time/month	3
		sometimes	2
		Rarely or never	1
	Soy products	almost every day	5
		≥1 time/week	4
		≥1 time/month	3
		sometimes	2
		Rarely or never	1
	Nuts	almost every day	5
		≥1 time/week	4
		≥1 time/month	3
		sometimes	2
		Rarely or never	1
	Tea	almost every day	5
		≥1 time/week	4
		≥1 time/month	3
		sometimes	2
		Rarely or never	1
**Highly processed foods**	Refined grain	Yes	5
		No	1
	Pickled vegetables or kimchi	almost every day	5
		≥1 time/week	4
		≥1 time/month	3
		sometimes	2
		Rarely or never	1
	Sugars	almost every day	5
		≥1 time/week	4
		≥1 time/month	3
		sometimes	2
		Rarely or never	1
Animal-based Foods	Animal fat	Yes	5
		No	1
	Meat	almost every day	5
		≥1 time/week	4
		≥1 time/month	3
		sometimes	2

Food groups	Category	Frequency	Score
		Rarely or never	1
	Aquatic products	almost every day	5
		≥1 time/week	4
		≥1 time/month	3
		sometimes	2
		Rarely or never	1
	Eggs	almost every day	5
		≥1 time/week	4
		≥1 time/month	3
		sometimes	2
		Rarely or never	1
	Dairy products	almost every day	5
		≥1 time/week	4
		≥1 time/month	3
		sometimes	2
		Rarely or never	1

Appendix S2 Prevalence of chronic diseases

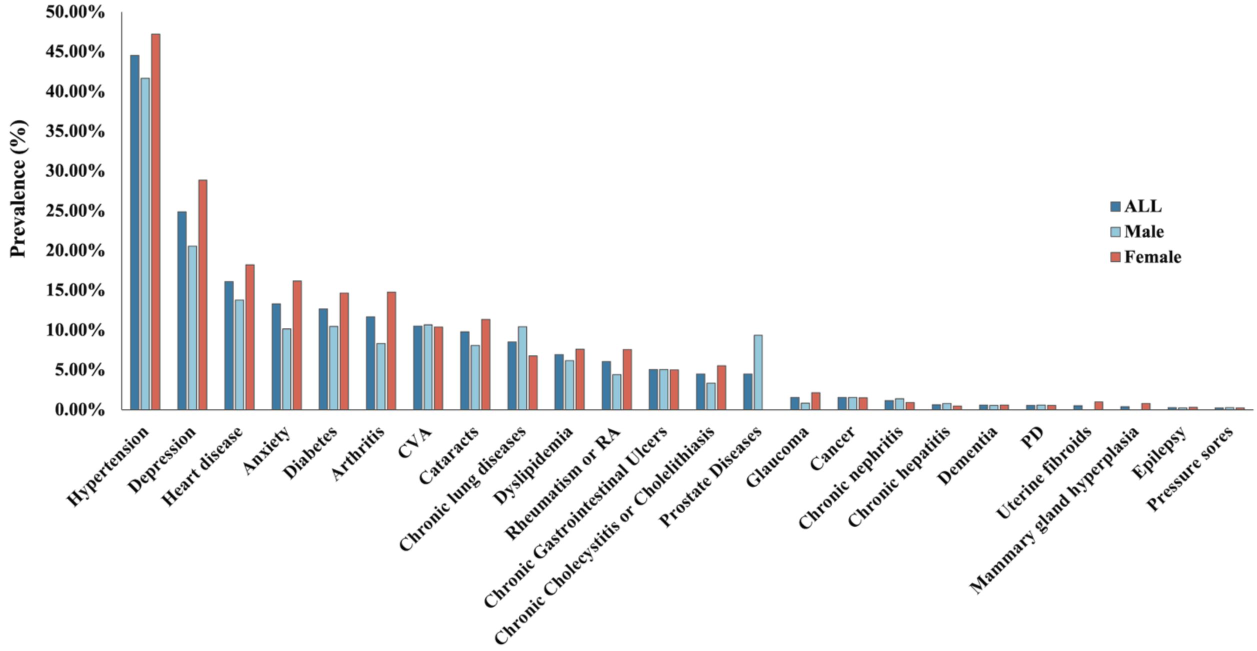

Appendix S3 Prevalence of multimorbidity in different provinces

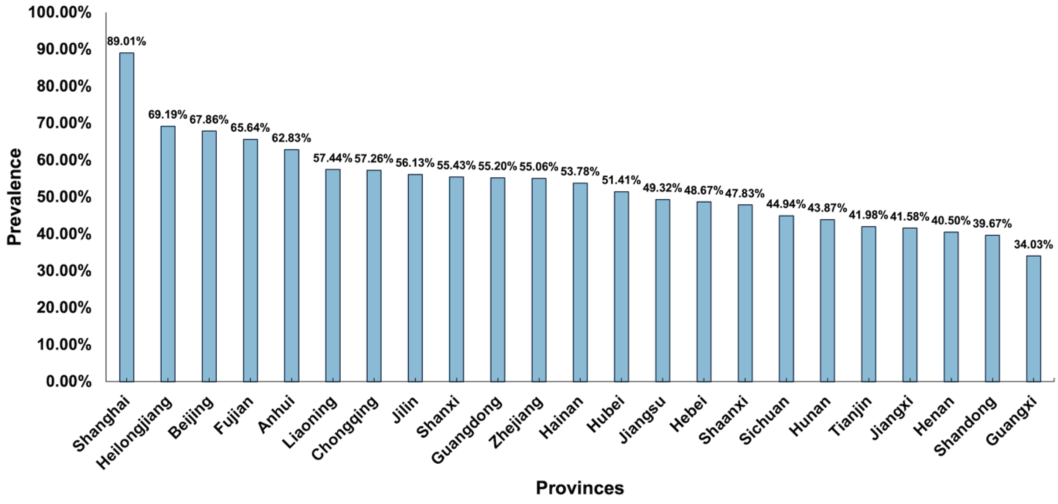

Appendix S4 Characteristics of the four Clusters

Table 1 Characteristics of the tumor-digestive disease cluster

Characteristics	N (n=3564)	Healthy (n=3383)	One disease (n=171)	Multimorbidity (n=10)	P^*
**Gender**					0.125
Male	1964 (55.1)	1857 (54.9)	104 (60.8)	3 (30.0)	
Female	1600 (44.9)	1526 (45.1)	67 (39.2)	7 (70.0)	
**Age(year)**	72.1±6.6	72.1±6.6	71.1±6.0	79.9±5.7	<0.001†
**Ethnicity**					0.220
Han	3200 (89.8)	3035 (89.7)	157 (91.8)	9 (90.0)	
Other	364 (10.2)	348 (10.3)	14 (8.2)	1 (10.0)	
**Residential Status**					0.890
Living alone	410 (11.5)	386 (11.4)	23 (13.5)	1 (10.0)	
Living with others	3154 (88.5)	2997 (88.6)	148 (86.5)	9 (90.0)	
**Marital status**					0.013
Currently married	2673 (75.0)	2524 (74.6)	144 (84.2)	5 (50.0)	
Divorced	862 (24.2)	832 (24.6)	25 (14.6)	5 (50.0)	
Never married	29 (0.8)	27 (0.8)	2 (1.2)	0 (0.0)	
**Heart rate (in beats / min)**	74.6±9.3	74.7±9.2	73.0±10.2	79.9±10.1	0.003†
**Weigh (Kg)**	58.0±11.4	58.2±11.6	55.4±8.5	48.0±7.5	<0.001†
**Height(cm)**	158.5±9.3	158.5±9.3	159.2±8.0	152.1±9.0	0.025†
**Waist circumference(cm)**	85.1±9.9	85.3±9.9	81.9±8.6	79.9±11.4	<0.001†
**Hip circumference(cm)**	93.1±8.8	93.2±8.8	90.6±9.1	90.7±6.9	0.002†
**BMI(Kg/m^2)**	23.1±4.7	23.1±4.7	21.8±2.7	21.0±4.3	<0.001†
**WHR**	0.9±0.1	0.9±0.1	0.9±0.1	0.9±0.1	0.123†
**Systolic(*mmHg*)**	134.7±17.1	134.8±16.9	132.4±20.0	142.0±18.7	0.011†
**Diastolic(*mmHg*)**	79.6±9.2	79.5±9.2	80.6±9.5	76.2±9.4	0.377†
**Staple food**					0.232
Rice	2106 (59.1)	1999 (59.1)	102 (59.6)	5 (50.0)	
Mixed grains	108 (3.0)	105 (3.1)	3 (1.8)	0 (0.0)	
Flour	709 (19.9)	666 (19.7)	42 (24.6)	1 (10.0)	
Rice and flour	627 (17.6)	599 (17.7)	24 (14.0)	4 (40.0)	
Other	14 (0.4)	14 (0.4)	0 (0.0)	0 (0.0)	
**Types of common cooking oil**					0.575
Vegetable oil	3167 (88.9)	3011 (89.0)	146 (85.4)	10 (100.0)	
Sesame oil	17 (0.5)	17 (0.5)	0 (0.0)	0 (0.0)	
Lard	366 (10.3)	341 (10.1)	25 (14.6)	0 (0.0)	
Animal fat	14 (0.4)	14 (0.4)	0 (0.0)	0 (0.0)	
**Drinking status**					0.690
Yes	1150 (32.3)	1086 (32.1)	61 (35.7)	3 (30.0)	
**Smoking status**					0.224
Yes	1276 (35.8)	1204 (35.6)	70 (40.9)	2 (20.0)	
**Physical exercise**					0.143
Yes	3288 (92.3)	3112 (92.0)	166 (97.1)	10 (100.0)	
**Vegetables**					0.215
Almost every day	2519 (70.7)	2378 (70.3)	132 (77.2)	9 (90.0)	
Often	866 (24.3)	836 (24.7)	29 (17.0)	1 (10.0)	
Sometimes	135 (3.8)	125 (3.7)	10 (5.8)	0 (0.0)	
Rarely or never	44 (1.2)	44 (1.3)	0 (0.0)	0 (0.0)	
**Fruits**					<0.001
Almost every day	818 (23.0)	795 (23.5)	20 (11.7)	3 (30.0)	

Characteristics	N (n=3564)	Healthy (n=3383)	One disease (n=171)	Multimorbidity (n=10)	*P*[*]
Often	1076 (30.2)	1055 (31.2)	21 (12.3)	0 (0.0)	
Sometimes	1064 (29.9)	978 (28.9)	82 (48.0)	4 (40.0)	
Rarely or never	606 (17.0)	555 (16.4)	48 (28.1)	3 (30.0)	
**Meat**					0.002
Almost every day	1568 (44.0)	1499 (44.3)	65 (38.0)	4 (40.0)	
≥1 time/week	1390 (39.0)	1326 (39.2)	60 (35.1)	4 (40.0)	
≥1 time/month	274 (7.7)	264 (7.8)	9 (5.3)	1 (10.0)	
Sometimes	150 (4.2)	135 (4.0)	14 (8.2)	1 (10.0)	
Rarely or never	182 (5.1)	159 (4.7)	23 (13.5)	0 (0.0)	
**Aquatic products**					<0.001
Almost every day	346 (9.7)	332 (9.8)	12 (7.0)	2 (20.0)	
≥1 time/week	1468 (41.2)	1391 (41.1)	73 (42.7)	4 (40.0)	
≥1 time/month	781 (21.9)	754 (22.3)	24 (14.0)	3 (30.0)	
Sometimes	506 (14.2)	487 (14.4)	19 (11.1)	0 (0.0)	
Rarely or never	463 (13.0)	419 (12.4)	43 (25.1)	1 (10.0)	
**Eggs**					0.100
Almost every day	1244 (34.9)	1198 (35.4)	42 (24.6)	4 (40.0)	
≥1 time/week	1390 (39.0)	1313 (38.8)	73 (42.7)	4 (40.0)	
≥1 time/month	438 (12.3)	408 (12.1)	29 (17.0)	1 (10.0)	
Sometimes	260 (7.3)	247 (7.3)	13 (7.6)	0 (0.0)	
Rarely or never	232 (6.5)	217 (6.4)	14 (8.2)	1 (10.0)	
**Soy products**					<0.001
Almost every day	415 (11.6)	406 (12.0)	7 (4.1)	2 (20.0)	
≥1 time/week	1430 (40.1)	1360 (40.2)	66 (38.6)	4 (40.0)	
≥1 time/month	830 (23.3)	792 (23.4)	37 (21.6)	1 (10.0)	
Sometimes	510 (14.3)	470 (13.9)	40 (23.4)	0 (0.0)	
Rarely or never	379 (10.6)	355 (10.5)	21 (12.3)	3 (30.0)	
**Pickled vegetables or kimchi**					0.040
Almost every day	474 (13.3)	447 (13.2)	25 (14.6)	2 (20.0)	
≥1 time/week	681 (19.1)	653 (19.3)	28 (16.4)	0 (0.0)	
≥1 time/month	471 (13.2)	440 (13.0)	27 (15.8)	4 (40.0)	
Sometimes	716 (20.1)	687 (20.3)	26 (15.2)	3 (30.0)	
Rarely or never	1222 (34.3)	1156 (34.2)	65 (38.0)	1 (10.0)	
**Sugars**					0.084
Almost every day	311 (8.7)	298 (8.8)	11 (6.4)	2 (20.0)	
≥1 time/week	656 (18.4)	612 (18.1)	42 (24.6)	2 (20.0)	
≥1 time/month	447 (12.5)	433 (12.8)	13 (7.6)	1 (10.0)	
Sometimes	685 (19.2)	660 (19.5)	25 (14.6)	0 (0.0)	
Rarely or never	1465 (41.1)	1380 (40.8)	80 (46.8)	5 (50.0)	
**Garlic**					0.008
Almost every day	809 (22.7)	771 (22.8)	35 (20.5)	3 (30.0)	
≥1 time/week	1053 (29.5)	995 (29.4)	56 (32.7)	2 (20.0)	
≥1 time/month	538 (15.1)	514 (15.2)	23 (13.5)	1 (10.0)	
Sometimes	517 (14.5)	504 (14.9)	10 (5.8)	3 (30.0)	
Rarely or never	647 (18.2)	599 (17.7)	47 (27.5)	1 (10.0)	
**Dairy products**					0.230
Almost every day	569 (16.0)	541 (16.0)	24 (14.0)	4 (40.0)	
≥1 time/week	501 (14.1)	477 (14.1)	23 (13.5)	1 (10.0)	
≥1 time/month	344 (9.7)	335 (9.9)	9 (5.3)	0 (0.0)	
Sometimes	519 (14.6)	491 (14.5)	27 (15.8)	1 (10.0)	

Characteristics	N (n=3564)	Healthy (n=3383)	One disease (n=171)	Multimorbidity (n=10)	*P*[*]
Rarely or never	1631 (45.8)	1539 (45.5)	88 (51.5)	4 (40.0)	
**Nuts**					<0.001
Almost every day	249 (7.0)	244 (7.2)	4 (2.3)	1 (10.0)	
≥1 time/week	552 (15.5)	535 (15.8)	17 (9.9)	0 (0.0)	
≥1 time/month	492 (13.8)	476 (14.1)	16 (9.4)	0 (0.0)	
Sometimes	798 (22.4)	758 (22.4)	38 (22.2)	2 (20.0)	
Rarely or never	1473 (41.3)	1370 (40.5)	96 (56.1)	7 (70.0)	
**Tea**					0.101
Almost every day	761 (21.4)	721 (21.3)	36 (21.1)	4 (40.0)	
≥1 time/week	124 (3.5)	115 (3.4)	9 (5.3)	0 (0.0)	
≥1 time/month	67 (1.9)	64 (1.9)	3 (1.8)	0 (0.0)	
Sometimes	134 (3.8)	132 (3.9)	2 (1.2)	0 (0.0)	
Rarely or never	2478 (69.5)	2351 (69.5)	121 (70.8)	6 (60.0)	
**Medication status**					<0.001
Yes	249 (7.0)	139 (4.1)	101 (59.1)	9 (90.0)	
**Nutritional supplement usage**					0.694
Yes	296 (8.3)	281 (8.3)	15 (8.8)	0 (0.0)	
**Cognitive impairment**					0.340
Yes	41 (1.2)	41 (1.2)	0 (0.0)	0 (0.0)	
**Public old age insurance**					0.030
Yes	1935 (54.3)	1854 (54.8)	75 (43.9)	6 (60.0)	
**Education level**					0.454[†]
Illiterate	877 (24.6)	839 (24.8)	34 (19.9)	4 (40.0)	
Primary education	1864 (52.3)	1756 (51.9)	103 (60.2)	5 (50.0)	
Secondary education and above	823 (23.1)	788 (23.3)	34 (19.9)	1 (10.0)	
**Residential area**					0.039
City	499 (14.0)	480 (14.2)	18 (10.5)	1 (10.0)	
Town	1062 (29.8)	1022 (30.2)	38 (22.2)	2 (20.0)	
Rural	2003 (56.2)	1881 (55.6)	115 (67.3)	7 (70.0)	

***** Pearson $\chi 2$ trend test, except † the Wilcoxon Rank Sum Test

Table 2 Characteristics of the metabolic-circulatory disease cluster

Characteristics	N (n=6541)	Healthy (n=3383)	One disease (n=2043)	Multimorbidity (n=1115)	*P**
**Gender**					<0.001
Male	3375 (51.6)	1857 (54.9)	1043 (50.6)	484 (43.4)	
Female	3166 (48.4)	1526 (45.1)	1009 (49.4)	631 (56.6)	
**Age(year)**	72.4±6.5	72.1±6.6	72.6±6.5	72.7±6.2	<0.001†
**Ethnicity**					<0.001
Han	6044 (92.4)	3035 (89.7)	1929 (94.4)	1080 (96.9)	
Other	497 (7.6)	348 (10.3)	114 (5.6)	35 (3.1)	
**Residential Status**					<0.001
Living alone	805 (12.3)	386 (11.4)	266 (13.0)	962 (86.3)	
Living with others	5736 (87.7)	2997 (88.6)	1777 (87.0)	152 (13.7)	
**Marital status**					<0.001
Currently married	4814 (73.6)	2524 (74.6)	1481 (72.5)	809 (72.6)	
Divorced	1681 (25.7)	832 (24.6)	545 (26.7)	304 (27.2)	
Never married	46 (0.7)	27 (0.8)	16 (0.8)	2 (0.2)	
**Heart rate (in beats / min)**	75.1±9.9	74.7±9.2	75.5±10.1	75.6±11.1	0.003†
**Weigh (Kg)**	60.0±11.6	58.2±11.6	61.0±11.6	63.7±10.7	<0.001†
**Height(cm)**	158.7±9.5	158.5±9.3	158.6±10.3	159.5±8.7	<0.001†
**Waist circumference(cm)**	87.1±10.2	85.3±9.9	88.0±10.2	90.9±9.6	<0.001†
**Hip circumference(cm)**	94.7±9.0	93.2±8.8	95.2±8.9	98.2±8.9	<0.001†
**BMI(Kg/m^2)**	23.9±5.4	23.1±4.7	24.4±6.7	25.0±3.8	<0.001†
**WHR**	0.9±0.1	0.9±0.1	0.9±0.1	0.9±0.1	<0.001†
**Systolic(*mmHg*)**	139.8±18.7	134.8±16.9	145.9±19.2	143.9±18.7	<0.001†
**Diastolic(*mmHg*)**	81.4±10.0	79.5±9.2	84.1±10.6	82.0±10.0	<0.001†
**Staple food**					<0.001
Rice	3702 (56.6)	1999 (59.1)	1162 (56.9)	541 (48.5)	
Mixed grains	222 (3.4)	105 (3.1)	59 (2.9)	58 (5.2)	
Flour	1315 (20.1)	666 (19.7)	402 (19.7)	246 (22.1)	
Rice and flour	1269 (19.4)	599 (17.7)	411 (20.1)	259 (23.3)	
Other	33 (0.5)	14 (0.4)	8 (0.4)	11 (0.9)	
**Types of common cooking oil**					<0.001
Vegetable oil	5978 (91.4)	3011 (89.0)	1890 (92.5)	1078 (96.7)	
Sesame oil	39 (0.6)	17 (0.5)	16 (0.8)	6 (0.5)	
Lard	504 (7.7)	341 (10.1)	133 (6.5)	30 (2.7)	
Animal fat	20 (0.3)	14 (0.4)	4 (0.2)	2 (0.1)	
**Drinking status**					<0.001
Yes	2060 (31.5)	1086 (32.1)	674 (33.0)	300 (26.9)	
**Smoking status**					<0.001
Yes	2224 (34.0)	1204 (35.6)	672 (32.9)	348 (31.2)	
**Physical exercise**					<0.001
Yes	5978 (91.4)	3112 (92.0)	1890 (92.5)	977 (87.6)	
**Vegetables**					<0.001
Almost every day	4657 (71.2)	2378 (70.3)	1483 (72.6)	796 (71.4)	
Often	1589 (24.3)	836 (24.7)	484 (23.7)	269 (24.1)	
Sometimes	222 (3.4)	125 (3.7)	59 (2.9)	38 (3.4)	
Rarely or never	72 (1.1)	44 (1.3)	16 (0.8)	12 (1.0)	
**Fruits**					<0.001
Almost every day	1688 (25.5)	795 (23.5)	601 (29.4)	272 (24.4)	
Often	1917 (29.3)	1055 (31.2)	529 (25.9)	332 (29.8)	

Characteristics	N (n=6541)	Healthy (n=3383)	One disease (n=2043)	Multimorbidity (n=1115)	*P*[*]
Sometimes	1851 (28.3)	978 (28.9)	588 (28.8)	285 (25.5)	
Rarely or never	1105 (16.9)	555 (16.4)	325 (15.9)	226 (20.2)	
**Meat**					<0.001
Almost every day	2662 (40.7)	1499 (44.3)	817 (40.0)	346 (31.0)	
≥1 time/week	2649 (40.5)	1326 (39.2)	829 (40.6)	494 (44.3)	
≥1 time/month	576 (8.8)	264 (7.8)	188 (9.2)	124 (11.1)	
Sometimes	268 (4.1)	135 (4.0)	82 (4.0)	51 (4.6)	
Rarely or never	386 (5.9)	159 (4.7)	127 (6.2)	100 (9.0)	
**Aquatic products**					<0.001
Almost every day	661 (10.1)	332 (9.8)	223 (10.9)	106 (9.5)	
≥1 time/week	2786 (42.6)	1391 (41.1)	917 (44.9)	478 (42.9)	
≥1 time/month	1380 (21.1)	754 (22.3)	382 (18.7)	244 (21.9)	
Sometimes	870 (13.3)	487 (14.4)	229 (11.2)	154 (13.8)	
Rarely or never	844 (12.9)	419 (12.4)	292 (14.3)	133 (11.9)	
**Eggs**					<0.001
Almost every day	2329 (35.6)	1198 (35.4)	721 (35.3)	409 (36.7)	
≥1 time/week	2499 (38.2)	1313 (38.8)	782 (38.3)	403 (36.2)	
≥1 time/month	791 (12.1)	408 (12.1)	245 (12.0)	138 (12.4)	
Sometimes	419 (6.4)	247 (7.3)	114 (5.6)	57 (5.1)	
Rarely or never	504 (7.7)	217 (6.4)	180 (8.8)	107 (9.6)	
**Soy products**					<0.001
Almost every day	857 (13.1)	406 (12.0)	298 (14.6)	153 (13.7)	
≥1 time/week	2662 (40.7)	1360 (40.2)	848 (41.5)	454 (40.7)	
≥1 time/month	1511 (23.1)	792 (23.4)	447 (21.9)	272 (24.4)	
Sometimes	831 (12.7)	470 (13.9)	241 (11.8)	120 (10.7)	
Rarely or never	680 (10.4)	355 (10.5)	208 (10.2)	117 (10.5)	
**Pickled vegetables or kimchi**					0.003
Almost every day	903 (13.8)	447 (13.2)	294 (14.4)	161 (14.5)	
≥1 time/week	1204 (18.4)	653 (19.3)	362 (17.7)	189 (16.9)	
≥1 time/month	890 (13.6)	440 (13.0)	270 (13.2)	180 (16.1)	
Sometimes	1230 (18.8)	687 (20.3)	349 (17.1)	193 (17.3)	
Rarely or never	2316 (35.4)	1156 (34.2)	768 (37.6)	391 (35.1)	
**Sugars**					<0.001
Almost every day	536 (8.2)	298 (8.8)	168 (8.2)	71 (6.4)	
≥1 time/week	1033 (15.8)	612 (18.1)	302 (14.8)	119 (10.7)	
≥1 time/month	798 (12.2)	433 (12.8)	249 (12.2)	116 (10.4)	
Sometimes	1256 (19.2)	660 (19.5)	411 (20.1)	185 (16.6)	
Rarely or never	2917 (44.6)	1380 (40.8)	913 (44.7)	624 (56.0)	
**Garlic**					<0.001
Almost every day	1576 (24.1)	771 (22.8)	525 (25.7)	280 (25.1)	
≥1 time/week	1799 (27.5)	995 (29.4)	537 (26.3)	266 (23.9)	
≥1 time/month	1119 (17.1)	514 (15.2)	374 (18.3)	231 (20.7)	
Sometimes	968 (14.8)	504 (14.9)	278 (13.6)	186 (16.7)	
Rarely or never	1079 (16.5)	599 (17.7)	329 (16.1)	151 (13.6)	
**Dairy products**					<0.001
Almost every day	1243 (19.0)	541 (16.0)	447 (21.9)	254 (22.8)	
≥1 time/week	903 (13.8)	477 (14.1)	270 (13.2)	156 (14.0)	
≥1 time/month	654 (10.0)	335 (9.9)	178 (8.7)	141 (12.7)	
Sometimes	929 (14.2)	491 (14.5)	292 (14.3)	146 (13.1)	
Rarely or never	2813 (43.0)	1539 (45.5)	856 (41.9)	418 (37.5)	
**Nuts**					0.239

Characteristics	N (n=6541)	Healthy (n=3383)	One disease (n=2043)	Multimorbidity (n=1115)	*P**
Almost every day	530 (8.1)	244 (7.2)	198 (9.7)	88 (7.9)	
≥1 time/week	1020 (15.6)	535 (15.8)	337 (16.5)	148 (13.3)	
≥1 time/month	975 (14.9)	476 (14.1)	304 (14.9)	194 (17.4)	
Sometimes	1439 (22.0)	758 (22.4)	435 (21.3)	246 (22.1)	
Rarely or never	2577 (39.4)	1370 (40.5)	768 (37.6)	439 (39.4)	
**Tea**					<0.001
Almost every day	1419 (21.7)	721 (21.3)	468 (22.9)	231 (20.7)	
≥1 time/week	321 (4.9)	115 (3.4)	114 (5.6)	91 (8.2)	
≥1 time/month	124 (1.9)	64 (1.9)	39 (1.9)	21 (1.9)	
Sometimes	222 (3.4)	132 (3.9)	63 (3.1)	27 (2.4)	
Rarely or never	4454 (68.1)	2351 (69.5)	1359 (66.5)	745 (66.8)	
**Medication status**					<0.001
Yes	2885 (44.1)	139 (4.1)	1679 (82.2)	1066 (95.6)	
**Nutritional supplement usage**					<0.001
Yes	628 (9.6)	281 (8.3)	210 (10.3)	137 (12.2)	
**Cognitive impairment**					<0.001
Yes	59 (0.9)	41 (1.2)	14 (0.7)	4 (0.3)	
**Public old age insurance**					<0.001
Yes	3656 (55.9)	1854 (54.8)	1128 (55.2)	675 (60.5)	
**Education level**					0.523†
Illiterate	1511 (23.1)	839 (24.8)	458 (22.4)	310 (27.8)	
Primary education	3362 (51.4)	1756 (51.9)	1066 (52.2)	540 (48.4)	
Secondary education and above	1668 (25.5)	788 (23.3)	519 (25.4)	265 (23.8)	
**Residential area**					<0.001
City	1217 (18.6)	480 (14.2)	419 (20.5)	318 (28.5)	
Town	1923 (29.4)	1022 (30.2)	611 (29.9)	290 (26.0)	
Rural	3401 (52.0)	1881 (55.6)	1013 (49.6)	507 (45.5)	

***** Pearson $\chi 2$ trend test, except † the Wilcoxon Rank Sum Test

Table 3 Characteristics of the metal-psychological disease cluster

Characteristics	N (n=4327)	Healthy (n=3383)	One disease (n=621)	Multimorbidity (n=323)	P^*
**Gender**					<0.001
Male	2250 (52.0)	1857 (54.9)	282 (45.4)	111(34.4)	
Female	2077 (48.0)	1526 (45.1)	339 (54.6)	212(65.6)	
**Age(year)**	72.4±6.9	72.1±6.6	74.0±7.7	72.4±7.3	<0.001†
**Ethnicity**					0.034
Han	3881 (89.7)	3035 (89.7)	545 (87.8)	301 (93.2)	
Other	446 (10.3)	348 (10.3)	76 (12.2)	22 (6.8)	
**Residential Status**					<0.001
Living alone	557 (12.9)	386 (11.4)	515 (82.9)	65 (20.1)	
Living with others	3770 (87.1)	2997 (88.6)	106 (17.1)	258 (79.9)	
**Marital status**					<0.001
Currently married	3133 (72.4)	2524 (74.6)	404 (65.1)	205 (63.5)	
Divorced	1156 (26.7)	832 (24.6)	211 (34.0)	113 (35.0)	
Never married	38 (0.9)	27 (0.8)	6 (1.0)	5 (1.5)	
**Heart rate (in beats / min)**	75.0±9.4	74.7±9.2	76.6±10.0	74.6±9.6	<0.001†
**Weigh (Kg)**	57.3±12.1	58.2±11.6	53.5±13.6	55.5±12.5	<0.001†
**Height(cm)**	157.7±9.8	158.5±9.3	154.5±10.2	154.7±11.3	<0.001†
**Waist circumference(cm)**	84.7±9.9	85.3±9.9	82.0±8.9	84.3±10.4	<0.001†
**Hip circumference(cm)**	92.7±8.7	93.2±8.8	90.6±7.6	91.9±9.1	<0.001†
**BMI(Kg/m^2)**	23.1±5.5	23.1±4.7	22.5±6.1	23.6±9.8	<0.001†
**WHR**	0.9±0.1	0.9±0.1	0.9±0.1	0.9±0.1	<0.001†
**Systolic(*mmHg*)**	134.7±17.1	134.8±16.9	135.6±18.4	132.4±15.7	0.037†
**Diastolic(*mmHg*)**	79.3±9.4	79.5±9.2	78.9±10.6	78.1±8.6	0.011†
**Staple food**					<0.001
Rice	2657 (61.4)	1999 (59.1)	433 (69.7)	225 (69.7)	
Mixed grains	121 (2.8)	105 (3.1)	10 (1.6)	6 (1.9)	
Flour	814 (18.8)	666 (19.7)	101 (16.3)	47 (14.6)	
Rice and flour	708 (16.4)	599 (17.7)	70 (11.3)	39 (12.1)	
Other	27 (0.6)	14 (0.4)	7 (1.1)	6 (1.9)	
**Types of common cooking oil**					<0.001
Vegetable oil	3776 (87.3)	3011 (89.0)	500 (80.5)	265 (82.0)	
Sesame oil	20 (0.5)	17 (0.5)	3 (0.5)	0 (0.0)	
Lard	515 (11.9)	341 (10.1)	116 (18.7)	58 (18.0)	
Animal fat	16 (0.4)	14 (0.4)	2 (0.3)	0 (0.0)	
**Drinking status**					0.003
Yes	1338 (30.9)	1086 (32.1)	158 (25.4)	94 (29.1)	
**Smoking status**					<0.001
Yes	1492 (34.5)	1204 (35.6)	206 (33.2)	82 (25.4)	
**Physical exercise**					<0.001
Yes	3959 (91.5)	3112 (92.0)	571 (91.9)	276 (85.4)	
**Vegetables**					<0.001
Almost every day	2951 (68.2)	2378 (70.3)	380 (61.2)	193 (59.8)	
Often	1084 (25.1)	836 (24.7)	171 (27.5)	77 (23.8)	
Sometimes	205 (4.7)	125 (3.7)	44 (7.1)	36 (11.1)	
Rarely or never	87 (2.0)	44 (1.3)	26 (4.2)	17 (5.3)	
**Fruits**					<0.001
Almost every day	902 (20.8)	795 (23.5)	70 (11.3)	37 (11.5)	
Often	1270 (29.4)	1055 (31.2)	152 (24.5)	63 (19.5)	
Sometimes	1362 (31.5)	978 (28.9)	259 (41.7)	125 (38.7)	
Rarely or never	793 (18.3)	555 (16.4)	140 (22.5)	98 (30.3)	

Characteristics	N (n=4327)	Healthy (n=3383)	One disease (n=621)	Multimorbidity (n=323)	*P*[*]
**Meat**					<0.001
Almost every day	1835 (42.4)	1499 (44.3)	232 (37.4)	104 (32.2)	
≥1 time/week	1697 (39.2)	1326 (39.2)	248 (39.9)	123 (38.1)	
≥1 time/month	385 (8.9)	264 (7.8)	72 (11.6)	49 (15.2)	
Sometimes	178 (4.1)	135 (4.0)	35 (5.6)	8 (2.5)	
Rarely or never	232 (5.4)	159 (4.7)	34 (5.5)	39 (12.1)	
**Aquatic products**					<0.001
Almost every day	407 (9.4)	332 (9.8)	43 (6.9)	32 (9.9)	
≥1 time/week	1719 (39.7)	1391 (41.1)	202 (32.5)	126 (39.0)	
≥1 time/month	951 (22.0)	754 (22.3)	135 (21.7)	62 (19.2)	
Sometimes	632 (14.6)	487 (14.4)	104 (16.7)	41 (12.7)	
Rarely or never	618 (14.3)	419 (12.4)	137 (22.1)	62 (19.2)	
**Eggs**					<0.001
Almost every day	1387 (32.1)	1198 (35.4)	119 (19.2)	70 (21.7)	
≥1 time/week	1696 (39.2)	1313 (38.8)	245 (39.5)	138 (42.7)	
≥1 time/month	567 (13.1)	408 (12.1)	113 (18.2)	46 (14.2)	
Sometimes	339 (7.8)	247 (7.3)	67 (10.8)	25 (7.7)	
Rarely or never	338 (7.8)	217 (6.4)	77 (12.4)	44 (13.6)	
**Soy products**					<0.001
Almost every day	481 (11.1)	406 (12.0)	56 (9.0)	19 (5.9)	
≥1 time/week	1719 (39.7)	1360 (40.2)	235 (37.8)	124 (38.4)	
≥1 time/month	995 (23.0)	792 (23.4)	142 (22.9)	61 (18.9)	
Sometimes	628 (14.5)	470 (13.9)	100 (16.1)	58 (18.0)	
Rarely or never	504 (11.6)	355 (10.5)	88 (14.2)	61 (18.9)	
**Pickled vegetables or kimchi**					0.294
Almost every day	559 (12.9)	447 (13.2)	67 (10.8)	45 (13.9)	
≥1 time/week	827 (19.1)	653 (19.3)	105 (16.9)	69 (21.4)	
≥1 time/month	572 (13.2)	440 (13.0)	89 (14.3)	43 (13.3)	
Sometimes	866 (20.0)	687 (20.3)	121 (19.5)	58 (18.0)	
Rarely or never	1503 (34.7)	1156 (34.2)	239 (38.5)	108 (33.4)	
**Sugars**					0.016
Almost every day	365 (8.4)	298 (8.8)	33 (5.3)	34 (10.5)	
≥1 time/week	787 (18.2)	612 (18.1)	111 (17.9)	64 (19.8)	
≥1 time/month	560 (12.9)	433 (12.8)	83 (13.4)	44 (13.6)	
Sometimes	826 (19.1)	660 (19.5)	125 (20.1)	41 (12.7)	
Rarely or never	1789 (41.3)	1380 (40.8)	269 (43.3)	140 (43.3)	
**Garlic**					0.470
Almost every day	990 (22.9)	771 (22.8)	144 (23.2)	75 (23.2)	
≥1 time/week	1257 (29.1)	995 (29.4)	165 (26.6)	97 (30.0)	
≥1 time/month	646 (14.9)	514 (15.2)	91 (14.7)	41 (12.7)	
Sometimes	665 (15.4)	504 (14.9)	101 (16.3)	60 (18.6)	
Rarely or never	769 (17.8)	599 (17.7)	120 (19.3)	50 (15.5)	
**Dairy products**					<0.001
Almost every day	622 (14.4)	541 (16.0)	46 (7.4)	35 (10.8)	
≥1 time/week	592 (13.7)	477 (14.1)	74 (11.9)	41 (12.7)	
≥1 time/month	428 (9.9)	335 (9.9)	47 (7.6)	46 (14.2)	
Sometimes	660 (15.3)	491 (14.5)	128 (20.6)	41 (12.7)	
Rarely or never	2025 (46.8)	1539 (45.5)	326 (52.5)	160 (49.5)	
**Nuts**					<0.001
Almost every day	288 (6.7)	244 (7.2)	22 (3.5)	22 (6.8)	
≥1 time/week	615 (14.2)	535 (15.8)	43 (6.9)	37 (11.5)	

Characteristics	N (n=4327)	Healthy (n=3383)	One disease (n=621)	Multimorbidity (n=323)	*P*[*]
≥1 time/month	570 (13.2)	476 (14.1)	50 (8.1)	44 (13.6)	
Sometimes	966 (22.3)	758 (22.4)	160 (25.8)	48 (14.9)	
Rarely or never	1888 (43.6)	1370 (40.5)	346 (55.7)	172 (53.3)	
**Tea**					<0.001
Almost every day	841 (19.4)	721 (21.3)	67 (10.8)	53 (16.4)	
≥1 time/week	151 (3.5)	115 (3.4)	29 (4.7)	7 (2.2)	
≥1 time/month	83 (1.9)	64 (1.9)	11 (1.8)	8 (2.5)	
Sometimes	173 (4.0)	132 (3.9)	25 (4.0)	16 (5.0)	
Rarely or never	3079 (71.2)	2351 (69.5)	489 (78.7)	239 (74.0)	
**Medication status**					<0.001
Yes	189 (4.4)	139 (4.1)	21 (3.4)	29 (9.0)	
**Nutritional supplement usage**					0.003
Yes	328 (7.6)	281 (8.3)	31 (5.0)	16 (5.0)	
**Cognitive impairment**					<0.001
Yes	70 (1.6)	41 (1.2)	11 (1.8)	18 (5.6)	
**Public old age insurance**					<0.001
Yes	2241 (51.8)	1854 (54.8)	240 (38.6)	147 (45.5)	
**Education level**					<0.001[†]
Illiterate	1188 (27.5)	839 (24.8)	223 (35.9)	126 (39.0)	
Primary education	2245 (51.9)	1756 (51.9)	336 (54.1)	153 (47.4)	
Secondary education and above	894 (20.7)	788 (23.3)	62 (10.0)	44 (13.6)	
**Residential area**					<0.001
City	538 (12.4)	480 (14.2)	33 (5.3)	25 (7.7)	
Town	1370 (31.7)	1022 (30.2)	243 (39.1)	105 (32.5)	
Rural	2419 (55.9)	1881 (55.6)	345 (55.6)	193 (59.8)	

***** Pearson $\chi 2$ trend test, except [†] the Wilcoxon Rank Sum Test

Table 4 Characteristics of the age-decline disease cluster

Characteristics	N (n=4118)	Healthy (n=3383)	One disease (n=638)	Multimorbidity (n=97)	P^*
**Gender**					<0.001
Male	2230 (54.2)	1857 (54.9)	341 (53.4)	32 (33.0)	
Female	1883 (45.8)	1526 (45.1)	297 (46.6)	65 (67.0)	
**Age(year)**	72.3±6.7	72.1±6.6	73.3±6.9	72.8±6.0	<0.001†
**Ethnicity**					0.075
Han	3710 (90.1)	3035 (89.7)	582 (91.2)	93 (95.9)	
Other	408 (9.9)	348 (10.3)	56 (8.8)	4 (4.1)	
**Residential Status**					0.460
Living alone	482 (11.7)	386 (11.4)	83 (13.0)	13 (13.4)	
Living with others	3636 (88.3)	2997 (88.6)	555 (87.0)	84 (86.6)	
**Marital status**					<0.001
Currently married	3042 (73.9)	2524 (74.6)	458 (71.8)	60 (61.9)	
Divorced	1030 (25.0)	832 (24.6)	162 (25.4)	36 (37.1)	
Never married	46 (1.1)	27 (0.8)	18 (2.8)	1 (1.0)	
**Heart rate (in beats / min)**	74.7±9.3	74.7±9.2	75.2±9.4	72.9±10.7	0.138†
**Weigh (Kg)**	57.8±11.5	58.2±11.6	56.4±11.4	53.9±9.9	<0.001†
**Height(cm)**	158.3±9.3	158.5±9.3	157.3±8.9	154.8±8.3	<0.001†
**Waist circumference(cm)**	85.2±10.0	85.3±9.9	84.8±10.2	82.6±8.4	0.015†
**Hip circumference(cm)**	93.0±8.7	93.2±8.8	92.2±8.3	91.9±8.3	0.009†
**BMI(Kg/m^2)**	23.1±4.8	23.1±4.7	22.8±5.2	22.4±3.3	0.009†
**WHR**	0.9±0.1	0.9±0.1	0.9±0.1	0.9±0.1	0.031†
**Systolic(*mmHg*)**	134.6±17.0	134.8±16.9	134.3±17.5	131.8±16.9	0.109†
**Diastolic(*mmHg*)**	79.4±9.3	79.5±9.2	78.6±9.4	79.5±11.0	0.022†
**Staple food**					0.007
Rice	2446 (59.4)	1999 (59.1)	376 (58.9)	71 (73.2)	
Mixed grains	121 (2.9)	105 (3.1)	13 (2.0)	3 (3.1)	
Flour	809 (19.6)	666 (19.7)	139 (21.8)	4 (4.1)	
Rice and flour	728 (17.7)	599 (17.7)	110 (17.2)	19 (19.6)	
Other	14 (0.3)	14 (0.4)	0 (0.0)	0 (0.0)	
**Types of common cooking oil**					0.004
Vegetable oil	3665 (89.0)	3011 (89.0)	577 (90.4)	77 (79.4)	
Sesame oil	19 (0.5)	17 (0.5)	2 (0.3)	0 (0.0)	
Lard	412 (10.0)	341 (10.1)	52 (8.2)	19 (19.6)	
Animal fat	22 (0.5)	14 (0.4)	7 (1.1)	1 (1.0)	
**Drinking status**					0.014
Yes	1349 (32.8)	1086 (32.1)	238 (37.3)	25 (25.8)	
**Smoking status**					0.003
Yes	1488 (36.1)	1204 (35.6)	260 (40.8)	24 (24.7)	
**Physical exercise**					0.134
Yes	3772 (91.6)	3112 (92.0)	572 (89.7)	88 (90.7)	
**Vegetables**					0.002
Almost every day	2875 (69.8)	2378 (70.3)	426 (66.8)	71 (73.2)	
Often	1015 (24.6)	836 (24.7)	157 (24.6)	22 (22.7)	
Sometimes	175 (4.2)	125 (3.7)	48 (7.5)	2 (2.1)	
Rarely or never	53 (1.3)	44 (1.3)	7 (1.1)	2 (2.1)	
**Fruits**					<0.001
Almost every day	932 (22.6)	795 (23.5)	106 (16.6)	31 (32.0)	
Often	1247 (30.3)	1055 (31.2)	168 (26.3)	24 (24.7)	
Sometimes	1210 (29.4)	978 (28.9)	208 (32.6)	24 (24.7)	

Characteristics	N (n=4118)	Healthy (n=3383)	One disease (n=638)	Multimorbidity (n=97)	P^*
Rarely or never	729 (17.7)	555 (16.4)	156 (24.5)	18 (18.6)	
**Meat**					0.003
Almost every day	1802 (43.8)	1499 (44.3)	245 (38.4)	58 (59.8)	
≥1 time/week	1617 (39.3)	1326 (39.2)	267 (41.8)	24 (24.7)	
≥1 time/month	325 (7.9)	264 (7.8)	51 (8.0)	10 (10.3)	
Sometimes	170 (4.1)	135 (4.0)	33 (5.2)	2 (2.1)	
Rarely or never	204 (5.0)	159 (4.7)	42 (6.6)	3 (3.1)	
**Aquatic products**					<0.001
Almost every day	389 (9.4)	332 (9.8)	46 (7.2)	11 (11.3)	
≥1 time/week	1663 (40.4)	1391 (41.1)	230 (36.1)	42 (43.3)	
≥1 time/month	911 (22.1)	754 (22.3)	135 (21.2)	22 (22.7)	
Sometimes	580 (14.1)	487 (14.4)	78 (12.2)	15 (15.5)	
Rarely or never	575 (14.0)	419 (12.4)	149 (23.4)	7 (7.2)	
**Eggs**					0.159
Almost every day	1466 (35.6)	1198 (35.4)	235 (36.8)	33 (34.0)	
≥1 time/week	1590 (38.6)	1313 (38.8)	243 (38.1)	34 (35.1)	
≥1 time/month	498 (12.1)	408 (12.1)	71 (11.1)	19 (19.6)	
Sometimes	301 (7.3)	247 (7.3)	44 (6.9)	10 (10.3)	
Rarely or never	263 (6.4)	217 (6.4)	45 (7.1)	1 (1.0)	
**Soy products**					0.005
Almost every day	479 (11.6)	406 (12.0)	54 (8.5)	19 (19.6)	
≥1 time/week	1639 (39.8)	1360 (40.2)	244 (38.2)	35 (36.1)	
≥1 time/month	975 (23.7)	792 (23.4)	162 (25.4)	21 (21.6)	
Sometimes	575 (14.0)	470 (13.9)	89 (13.9)	16 (16.5)	
Rarely or never	450 (10.9)	355 (10.5)	89 (13.9)	6 (6.2)	
**Pickled vegetables or kimchi**					0.002
Almost every day	560 (13.6)	447 (13.2)	92 (14.4)	21 (21.6)	
≥1 time/week	793 (19.3)	653 (19.3)	117 (18.3)	23 (23.7)	
≥1 time/month	529 (12.8)	440 (13.0)	84 (13.2)	5 (5.2)	
Sometimes	797 (19.4)	687 (20.3)	93 (14.6)	17 (17.5)	
Rarely or never	1439 (34.9)	1156 (34.2)	252 (39.5)	31 (32.0)	
**Sugars**					<0.001
Almost every day	365 (8.9)	298 (8.8)	51 (8.0)	16 (16.5)	
≥1 time/week	749 (18.2)	612 (18.1)	118 (18.5)	19 (19.6)	
≥1 time/month	537 (13.0)	433 (12.8)	75 (11.8)	29 (29.9)	
Sometimes	809 (19.6)	660 (19.5)	142 (22.3)	7 (7.2)	
Rarely or never	1658 (40.3)	1380 (40.8)	252 (39.5)	26 (26.8)	
**Garlic**					0.005
Almost every day	951 (23.1)	771 (22.8)	154 (24.1)	26 (26.8)	
≥1 time/week	1197 (29.1)	995 (29.4)	184 (28.8)	18 (18.6)	
≥1 time/month	627 (15.2)	514 (15.2)	101 (15.8)	12 (12.4)	
Sometimes	589 (14.3)	504 (14.9)	64 (10.0)	21 (21.6)	
Rarely or never	754 (18.3)	599 (17.7)	135 (21.2)	20 (20.6)	
**Dairy products**					0.014
Almost every day	669 (16.2)	541 (16.0)	104 (16.3)	24 (24.7)	
≥1 time/week	584 (14.2)	477 (14.1)	85 (13.3)	22 (22.7)	
≥1 time/month	410 (10.0)	335 (9.9)	67 (10.5)	8 (8.2)	
Sometimes	599 (14.5)	491 (14.5)	91 (14.3)	17 (17.5)	
Rarely or never	1856 (45.1)	1539 (45.5)	291 (45.6)	26 (26.8)	
**Nuts**					0.009
Almost every day	308 (7.5)	244 (7.2)	53 (8.3)	11 (11.3)	

Characteristics	N (n=4118)	Healthy (n=3383)	One disease (n=638)	Multimorbidity (n=97)	*P*[*]
≥1 time/week	648 (15.7)	535 (15.8)	90 (14.1)	23 (23.7)	
≥1 time/month	587 (14.3)	476 (14.1)	93 (14.6)	18 (18.6)	
Sometimes	909 (22.1)	758 (22.4)	127 (19.9)	24 (24.7)	
Rarely or never	1666 (40.5)	1370 (40.5)	275 (43.1)	21 (21.6)	
**Tea**					0.246
Almost every day	882 (21.4)	721 (21.3)	143 (22.4)	18 (18.6)	
≥1 time/week	145 (3.5)	115 (3.4)	23 (3.6)	7 (7.2)	
≥1 time/month	76 (1.8)	64 (1.9)	10 (1.6)	2 (2.1)	
Sometimes	153 (3.7)	132 (3.9)	15 (2.4)	6 (6.2)	
Rarely or never	2862 (69.5)	2351 (69.5)	447 (70.1)	64 (66.0)	
**Medication status**					<0.001
Yes	488 (11.9)	139 (4.1)	303 (47.5)	46 (47.4)	
**Nutritional supplement usage**					<0.001
Yes	378 (9.2)	281 (8.3)	74 (11.6)	23 (23.7)	
**Cognitive impairment**					0.678
Yes	47 (1.1)	41 (1.2)	5 (0.8)	1 (1.0)	
**Public old age insurance**					0.304
Yes	2262 (54.9)	1854 (54.8)	361 (56.6)	47 (48.5)	
**Education level**					0.256[†]
Illiterate	939 (22.8)	839 (24.8)	126 (19.7)	25 (25.8)	
Primary education	2170 (52.7)	1756 (51.9)	368 (57.7)	46 (47.4)	
Secondary education and above	1009 (24.5)	788 (23.3)	144 (22.6)	26 (26.8)	
**Residential area**					0.106
City	569 (13.8)	480 (14.2)	72 (11.3)	17 (17.5)	
Town	1238 (30.1)	1022 (30.2)	195 (30.6)	21 (21.6)	
Rural	2331 (56.1)	1881 (55.6)	371 (58.2)	59 (60.8)	

***** Pearson $\chi 2$ trend test, except † the Wilcoxon Rank Sum Test

Appendix S5 Score for various clusters

Food groups	Total	Healthy	One disease	Multimorbidity	P
**Tumor-Digestive Disease Cluster**					
Plant-based food	24.21(4.64)	24.30(4.65)	22.41(3.95)	24.19(4.46)	<0.001
Highly processed foods	9.78(2.27)	9.78(2.27)	9.82(2.36)	10.37(2.37)	0.957
Animal-based Foods	15.04(3.28)	15.08(3.24)	14.23(3.88)	15.56(3.68)	<0.001
**Metabolic-Circulatory Disease Cluster**					
Plant-based food	24.62(4.65)	24.30(4.65)	24.62(4.65)	24.91(4.52)	<0.001
Highly processed foods	9.64(2.28)	9.78(2.27)	9.64(2.28)	9.27(2.29)	<0.001
Animal-based Foods	15.01(3.39)	15.08(3.24)	15.01(3.39)	14.78(3.70)	0.193
**Mental-Psychological Disease Cluster**					
Plant-based food	23.80(4.73)	24.30(4.65)	21.97(4.36)	22.09(4.94)	<0.001
Highly processed foods	9.75(2.29)	9.78(2.27)	9.53(2.26)	9.90(2.52)	0.011
Animal-based Foods	14.87(3.32)	15.08(3.24)	14.03(3.28)	14.29(3.84)	<0.001
**Elderly-Degenerative Disease Cluster**					
Plant-based food	24.21(4.69)	24.30(4.65)	23.67(4.77)	24.56(5.14)	0.046
Highly processed foods	9.80(2.28)	9.78(2.27)	9.79(2.31)	10.64(2.19)	0.001
Animal-based Foods	15.04(3.27)	15.08(3.24)	14.60(3.36)	16.48(3.19)	<0.001

Appendix S6 Association Rules Extracted from the Cluster

Consequents	Antecedents	Support (%)	Confidence (%)	Lift
Tumor-Digestive Disease Cluster		***1.0***	***10.0***	
Chronic gastrointestinal ulcers	Cancer	1.387	14.356	3.299
Chronic gastrointestinal ulcers	Glaucoma	1.854	13.333	3.064
Chronic gastrointestinal ulcers	Chronic cholecystitis or cholelithiasis	3.872	15.071	3.463
Glaucoma	Cancer	1.387	12.376	6.677
Chronic cholecystitis or cholelithiasis	Cancer	1.387	13.366	3.452
Chronic cholecystitis or cholelithiasis	Glaucoma	1.854	15.556	4.018
Chronic cholecystitis or cholelithiasis	Chronic gastrointestinal ulcers	4.352	13.407	3.463
Metabolic-Circulatory Disease Cluster		***1.0***	***50.0***	
Hypertension	Diabetes, CVA	1.812	85.606	2.115
Hypertension	Diabetes, Heart disease	3.165	84.382	2.084
Hypertension	Dyslipidemia, diabetes	1.620	83.898	2.072
Hypertension	Dyslipidemia, Heart disease	2.135	79.421	1.962
Hypertension	Dyslipidemia, CVA	1.428	78.365	1.936
Hypertension	Diabetes	9.384	73.299	1.811
Hypertension	CVA, Heart disease	3.666	71.910	1.776
Hypertension	Dyslipidemia	4.847	70.822	1.749
Hypertension	CVA	10.489	61.846	1.528
Hypertension	Heart disease	16.249	61.808	1.527
Heart disease	Dyslipidemia, CVA	1.428	59.615	3.669
Heart disease	Dyslipidemia, Diabetes	1.620	55.932	3.442
Heart disease	Diabetes, CVA	1.812	53.788	3.310
Mental-Psychological Disease Cluster		***1.0***	***50.0***	
Depression	Anxiety	14.567	72.856	2.274
Elderly-Degenerative Disease Cluster		***1.0***	***30.0***	
Arthritis	Rheumatism or RA	5.224	44.809	4.294
Arthritis	Rheumatism or RA, Cataracts	1.270	54.054	5.180
Arthritis	Chronic lung diseases, Cataracts	2.114	33.442	3.205
Rheumatism or RA	Chronic lung diseases, Arthritis	1.785	32.308	6.184
Chronic lung diseases	Prostate diseases, Arthritis	1.044	34.211	3.423
Chronic lung diseases	Prostate diseases, Cataracts	1.634	31.513	3.153
Cataracts	Arthritis	10.435	30.395	2.318
Cataracts	Prostate diseases, Chronic lung diseases	1.222	42.135	3.213
Cataracts	Prostate diseases, Arthritis	1.043	40.132	3.061
Cataracts	Chronic lung diseases, Arthritis	1.785	39.615	3.021

CVA: Stroke and Cerebrovascular Diseases; RA: Rheumatoid Arthritis

Appendix S7. Variations of Multimorbidity Patterns by Region

The Multimorbidity Pattern in the Northern China Group

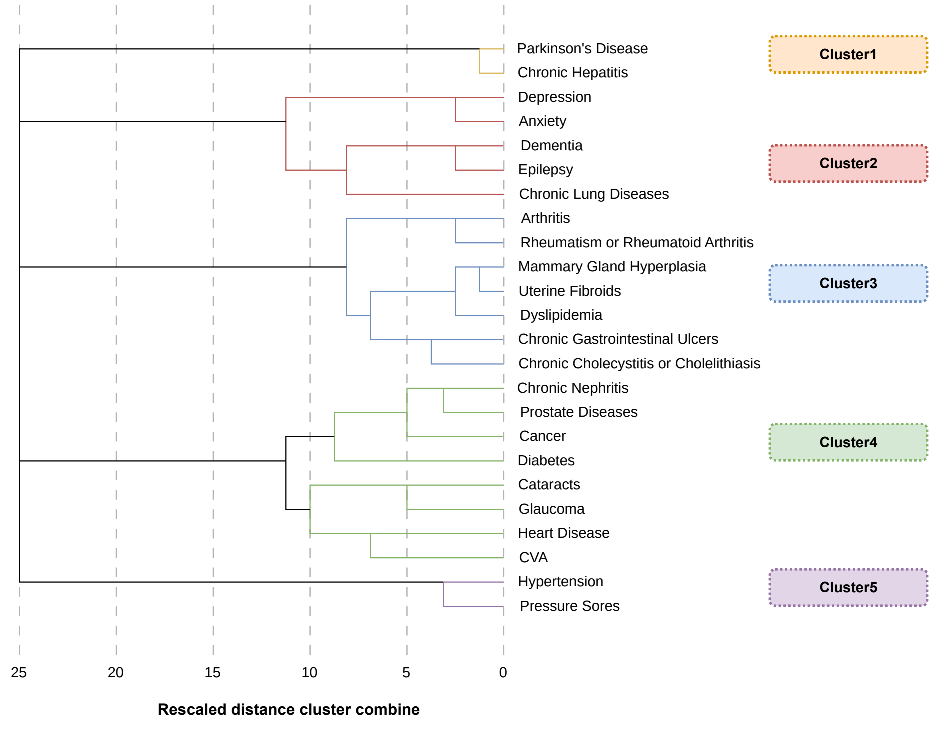

Figure 1. Results of Cluster Analysis

Table 1 Results of ARM

**Consequents**	**Antecedents**	**Support (%)**	**Confidence (%)**	**Lift**
Cluster 1		***1.0***	***10.0***	
Chronic hepatitis	Parkinson's disease	1.083	11.111	23.083
Cluster 2		1.0	50.0	
Depression	Chronic lung diseases, Anxiety	1.564	76.923	3.245
Depression	Anxiety	11.252	70.588	2.978
Depression	Dementia	2.647	52.273	2.205
Cluster 3		***1.0***	***30.0***	
Dyslipidemia	Chronic gastrointestinal ulcers, Arthritis	1.083	44.444	4.617
Dyslipidemia	Chronic cholecystitis or cholelithiasis, Arthritis	1.444	50.000	5.194
Arthritis	Chronic gastrointestinal ulcers	3.490	31.034	2.399
Arthritis	Chronic cholecystitis or cholelithiasis	4.633	31.169	2.409
Arthritis	Rheumatism or RA	4.994	53.012	4.098
Arthritis	Dyslipidemia	9.627	35.000	2.706
Arthritis	Chronic gastrointestinal ulcers, Dyslipidemia	1.023	47.059	3.638
Arthritis	Chronic cholecystitis or cholelithiasis, Dyslipidemia	1.143	63.158	4.882
Chronic cholecystitis or cholelithiasis	Chronic gastrointestinal ulcers, Arthritis	1.083	33.333	7.195
Cluster 4		***1.0***	***50.0***	
Cataracts	Chronic nephritis	1.986	54.545	2.444
Cataracts	Glaucoma	3.550	52.542	2.354
Cataracts	Chronic nephritis, Heart disease	1.203	70.000	3.136
Cataracts	Glaucoma, Heart disease	1.324	77.273	3.462
Diabetes	Chronic nephritis, Cataracts	1.083	50.000	3.033
Diabetes	Glaucoma, Heart disease	1.324	54.545	3.309
Heart disease	Chronic nephritis	1.986	60.606	2.094
Heart disease	Chronic nephritis, Cataracts	1.083	77.777	2.687
Heart disease	Glaucoma, Cataracts	1.865	54.839	1.895
Heart disease	Prostate diseases, CVA	2.347	51.282	1.772
Heart disease	CVA, Diabetes	3.069	72.549	2.507
Heart disease	CVA, Cataracts	4.452	55.405	1.914
Heart disease	Diabetes, Cataracts	5.295	56.818	1.963
Cluster 5				
None	None	/	/	/

CVA: Stroke and Cerebrovascular Diseases; RA: Rheumatoid Arthritis

The Multimorbidity Pattern in the Eastern China Group

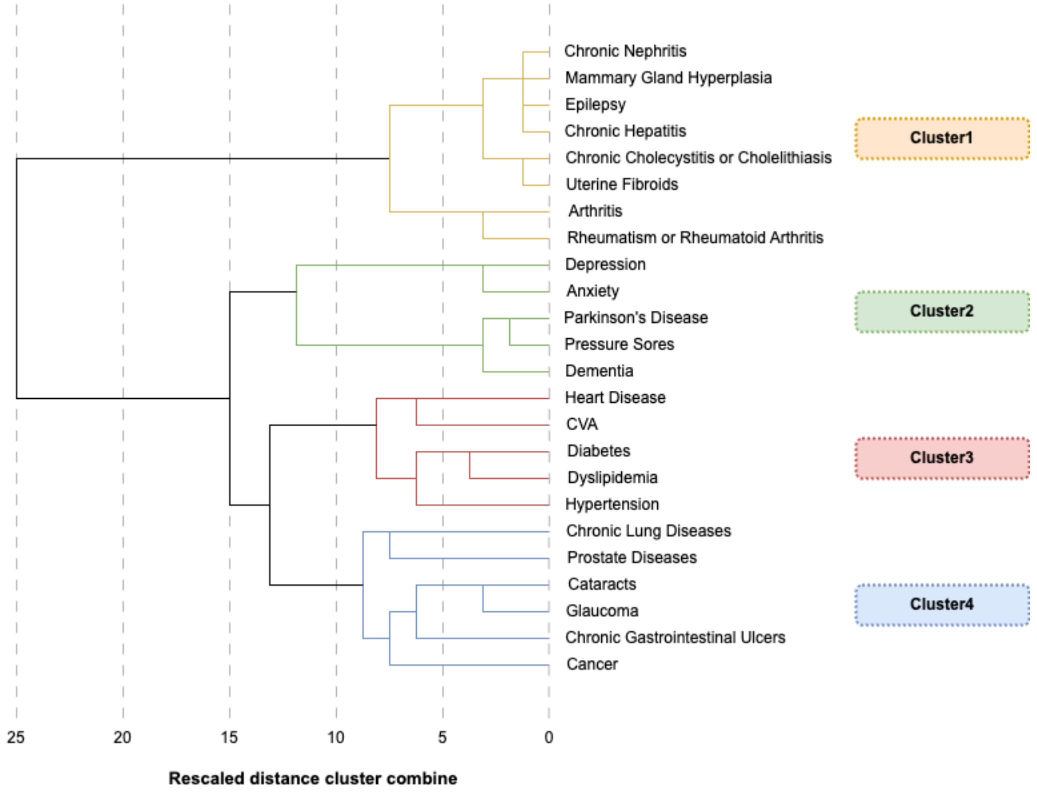

Figure 2. Results of Cluster Analysis

Table 2 Results of ARM

Consequents	Antecedents	Support (%)	Confidence (%)	Lift
Cluster 1		1.0	30.0	
Arthritis	Chronic cholecystitis or cholelithiasis	4.502	37.079	3.611
Arthritis	Rheumatism or RA	2.833	48.214	4.696
Cluster 2		1.0	30.0	
Anxiety	Depression	31.984	33.052	2.314
Anxiety	Dementia	1.956	37.931	2.656
Anxiety	Dementia, Depression	1.147	51.471	3.604
Depression	Dementia	1.956	58.621	1.833
Depression	Anxiety	14.281	74.026	2.314
Cluster 3		1.0	50.0	
Hypertension	Diabetes, CVA	2.428	90.278	1.990
Hypertension	Diabetes, Heart disease	3.625	86.512	1.907
Hypertension	Dyslipidemia, Diabetes	2.108	84.8	1.869
Hypertension	Dyslipidemia, CVA	1.737	83.495	1.840
Hypertension	Dyslipidemia, Heart disease	2.512	82.550	1.819
Hypertension	CVA, Heart disease	4.974	79.322	1.748
Hypertension	Diabetes	10.420	73.625	1.623
Hypertension	Dyslipidemia	5.952	72.805	1.605
Hypertension	CVA	12.544	66.801	1.472
Hypertension	Heart disease	18.412	65.934	1.453
Heart disease	Dyslipidemia, CVA	1.737	64.078	3.480
Heart disease	Diabetes, CVA	2.428	54.861	2.980
Heart disease	Dyslipidemia, Diabetes	2.108	53.6	2.911
Cluster 4		1.0	30.0	
Prostate diseases	Chronic lung diseases, Cataracts	2.209	31.298	5.492
Chronic gastrointestinal ulcers	Glaucoma, Cataracts	1.130	31.343	5.773
Cataracts	Chronic gastrointestinal ulcers	5.429	32.919	2.532
Cataracts	Prostate diseases	5.699	34.615	2.663
Chronic lung diseases	Prostate diseases, Cataracts	1.973	35.043	3.418
Cataracts	Prostate diseases, Chronic lung diseases	1.332	51.899	3.992
Cataracts	Glaucoma	2.006	56.303	4.331

CVA: Stroke and Cerebrovascular Diseases; RA: Rheumatoid Arthritis

The Multimorbidity Pattern in the Southern China Group

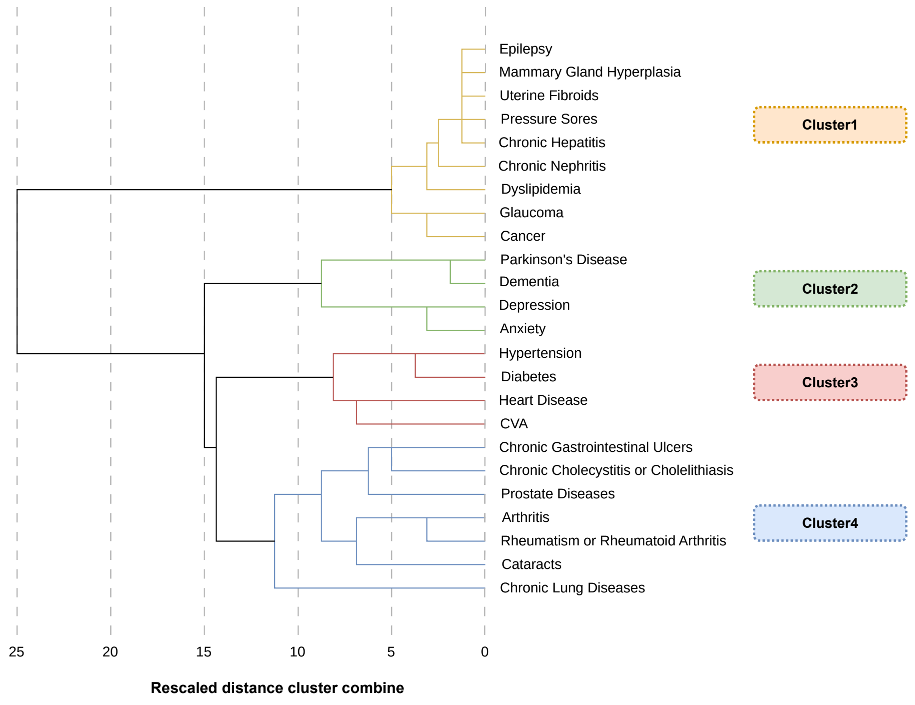

Figure 3. Results of Cluster Analysis

Table 3 Results of ARM

Consequents	Antecedents	Support (%)	Confidence (%)	Lift
Cluster 1		1.0	10.0	
Dyslipidemia	Chronic nephritis	1.034	18.75	6.803
Dyslipidemia	Glaucoma	1.077	18.0	6.531
Chronic hepatitis	Chronic nephritis	1.034	14.583	35.645
Cluster 2		1.0	30.0	
Depression	Anxiety	16.128	73.431	2.112
Depression	Dementia	1.744	69.136	1.988
Anxiety	Dementia, Depression	1.206	50.0	3.100
Anxiety	Dementia	1.744	41.975	2.603
Anxiety	Depression	34.776	34.056	2.112
Cluster 3		1.0	50.0	
Hypertension	Diabetes, Heart disease	1.637	81.579	2.314
Hypertension	Diabetes	7.106	72.727	2.063
Hypertension	CVA, Heart disease	1.443	61.194	1.736
Hypertension	Heart disease	9.432	58.219	1.652
Hypertension	CVA	6.654	55.663	1.579
Cluster 4		1.0	30.0	
Rheumatism or RA	Arthritis	11.025	32.617	3.924
Rheumatism or RA	Chronic lung diseases, Arthritis	1.723	48.75	5.865
Rheumatism or RA	Cataracts, Arthritis	2.649	34.146	4.108
Arthritis	Rheumatism or RA	8.312	43.264	3.924
Arthritis	Chronic lung diseases, Rheumatism or RA	1.357	61.905	5.615
Arthritis	Rheumatism or RA, Cataracts	1.938	46.667	4.233

CVA: Stroke and Cerebrovascular Diseases; RA: Rheumatoid Arthritis

The Multimorbidity Pattern in the Central China Group

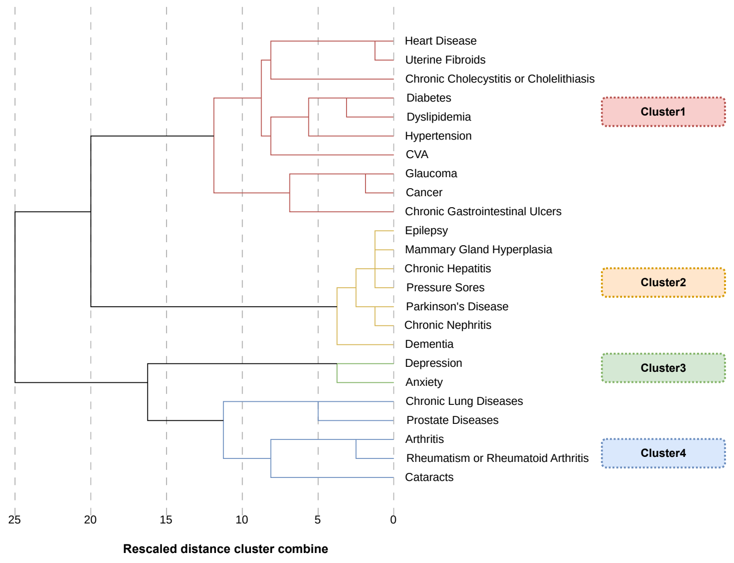

Figure 4. Results of Cluster Analysis

Table 4 Results of ARM

Consequents	Antecedents	Support (%)	Confidence (%)	Lift
Cluster 1		**1.0**	**50.000**	
CVA	Dyslipidemia, Heart disease	1.373	50.000	4.916
Heart disease	Dyslipidemia, CVA	1.073	64.000	4.189
Heart disease	Dyslipidemia, Hypertension	1.803	54.762	3.584
Hypertension	Glaucoma	1.803	50.000	1.469
Hypertension	Chronic cholecystitis or cholelithiasis	3.691	51.163	1.503
Hypertension	Dyslipidemia	2.790	64.615	1.899
Hypertension	Diabetes	6.223	75.862	2.229
Hypertension	CVA	10.172	53.165	1.562
Hypertension	Heart disease	15.279	50.281	1.477
Hypertension	Chronic cholecystitis or cholelithiasis, Heart disease	1.116	73.077	2.147
Hypertension	Dyslipidemia, CVA	1.073	64.000	1.880
Hypertension	Dyslipidemia, Heart disease	1.373	71.875	2.112
Hypertension	Diabetes, CVA	1.202	82.143	2.414
Hypertension	Diabetes, Heart disease	1.931	84.444	2.481
Hypertension	CVA, Heart disease	2.661	54.839	1.611
Cluster 2		**1.0**	**1.0**	
Chronic nephritis	Dementia	2.532	3.390	5.266
Pressure sores	Dementia	2.532	5.085	13.164
Parkinson's disease	Dementia	2.532	3.390	5.642
Chronic hepatitis	Dementia	2.532	1.695	5.642
Epilepsy	Dementia	2.532	5.085	16.925
Cluster 3		1.0	50.0	
Depression	Anxiety	14.549	69.912	2.141
Cluster 4		**1.0**	**30.0**	
Arthritis	Rheumatism or RA	5.322	39.516	5.004
Arthritis	Rheumatism or RA, Chronic lung diseases	1.245	58.621	7.423
Arthritis	Rheumatism or RA, Cataracts	1.159	59.259	7.504
Arthritis	Chronic lung diseases, Cataracts	1.674	38.462	4.870
Rheumatism or RA	Arthritis, Chronic lung diseases	1.502	48.571	9.127
Rheumatism or RA	Arthritis, Cataracts	1.674	41.026	7.709
Chronic lung diseases	Rheumatism or RA, Arthritis	2.103	34.694	3.593
Chronic lung diseases	Arthritis, Cataracts	1.674	38.462	3.983
Cataracts	Prostate Diseases, Chronic lung diseases	1.245	31.034	2.976
Cataracts	Rheumatism or RA, Arthritis	2.103	32.653	3.131
Cataracts	Arthritis, Chronic lung diseases	1.502	42.857	4.109

CVA: Stroke and Cerebrovascular Diseases; RA: Rheumatoid Arthritis

Appendix S8. Variations of Multimorbidity Patterns by Gender

The Multimorbidity Pattern in Elderly Chinese Men

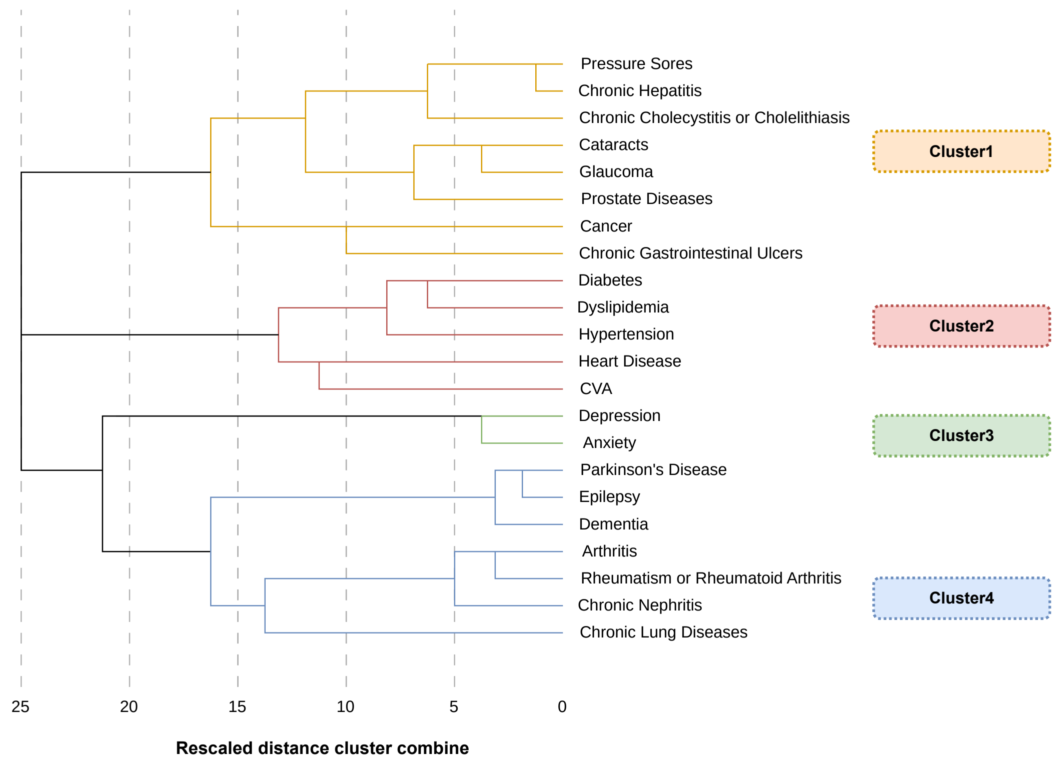

Figure 1. Results of Cluster Analysis

Table 1 Results of ARM

Consequents	Antecedents	Support (%)	Confidence (%)	Lift
Cluster 1		***1.0***	***30.0***	
Prostate diseases	Glaucoma	1.259	40.244	3.296
Prostate diseases	Cancer	1.612	33.333	2.730
Prostate diseases	Chronic cholecystitis or cholelithiasis	3.133	35.294	2.891
Prostate diseases	Chronic gastrointestinal ulcers	4.223	30.909	2.532
Prostate diseases	Cataracts	11.394	32.075	2.627
Prostate diseases	Chronic gastrointestinal ulcers，Cataracts	1.1057	59.722	4.892
Cataracts	Glaucoma	1.259	51.220	4.495
Cataracts	Chronic cholecystitis or cholelithiasis, Prostate diseases	1.106	48.611	4.266
Cataracts	Chronic gastrointestinal ulcers, Prostate diseases	1.305	50.588	4.440
Cluster 2		***1.0***	***50.0***	
Hypertension	Dyslipidemia, Diabetes	1.443	86.170	2.219
Hypertension	Diabetes, CVA	1.797	82.906	2.135
Hypertension	Diabetes, Heart disease	2.611	80.588	2.075
Hypertension	Dyslipidemia, CVA	1.305	77.647	1.999
Hypertension	Dyslipidemia, Heart disease	1.904	74.194	1.910
Hypertension	CVA, Heart disease	3.440	73.214	1.885
Hypertension	Diabetes	8.799	71.902	1.851
Hypertension	Dyslipidemia	4.592	69.900	1.800
Hypertension	Heart disease	14.803	59.336	1.528
Hypertension	CVA	11.640	59.103	1.522
Heart disease	Dyslipidemia, CVA	1.305	56.471	3.815
Heart disease	Diabetes, CVA	1.797	53.846	3.637
Cluster 3		***1.0***	***50.0***	
Depression	Anxiety	11.517	70.133	2.645
Cluster 4		***1.0***	***30.0***	
Arthritis	Chronic nephritis	1.152	41.333	4.975
Arthritis	Rheumatism or RA	3.931	41.016	4.937
Chronic lung diseases	Rheumatism or RA, Arthritis	1.612	33.333	2.660

CVA: Stroke and Cerebrovascular Diseases; RA: Rheumatoid Arthritis

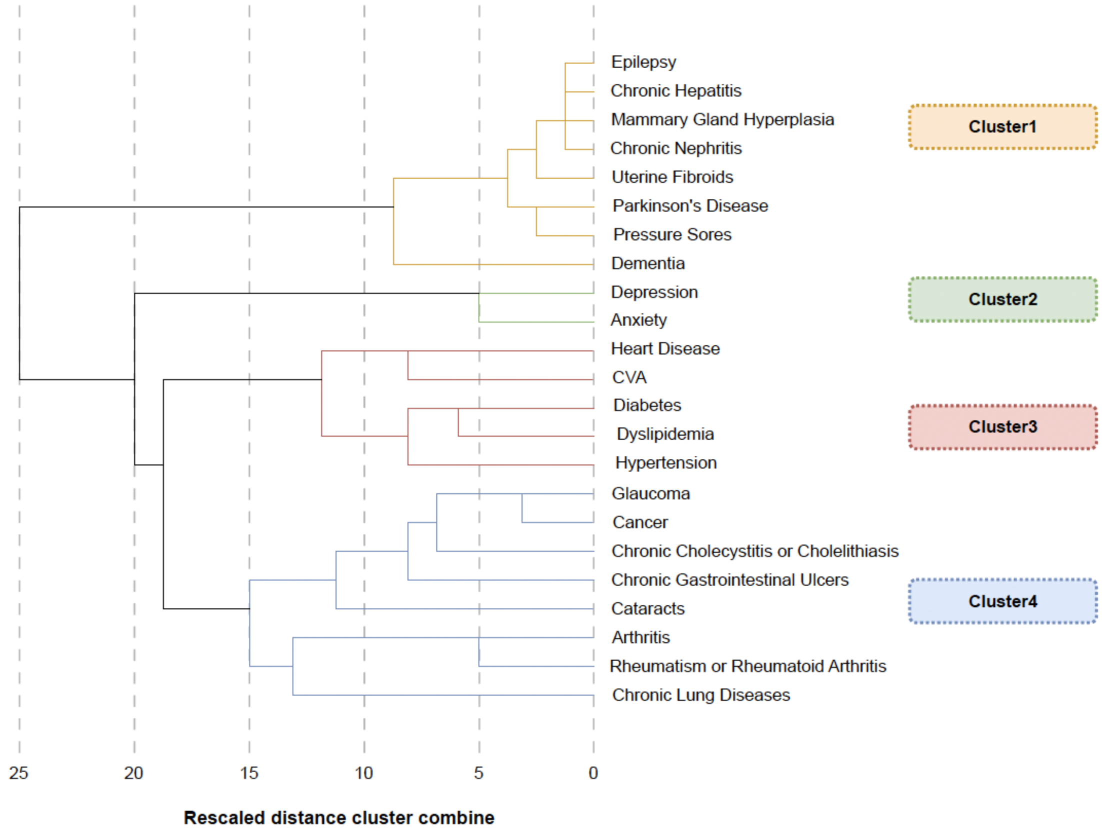

Figure 2. Results of Cluster Analysis

Table 2 Results of ARM

Consequents	Antecedents	Support (%)	Confidence (%)	Lift
Cluster 1		***1.0***	***1.0***	
Epilepsy	Dementia	2.582	3.846	14.753
Chronic hepatitis	Dementia	2.582	2.404	7.447
Parkinson's disease	Dementia	2.582	3.365	5.020
Mammary gland hyperplasia	Dementia	2.582	2.885	6.454
Pressure sores	Dementia	2.582	4.327	10.892
Chronic nephritis	Dementia	2.582	2.885	3.367
Uterine fibroids	Dementia	2.582	1.923	3.296
Cluster 2		***1.0***	***50.0***	
Hypertension	CVA, Diabetes	1.825	87.755	2.099
Hypertension	Diabetes, Heart disease	3.613	86.598	2.071
Hypertension	Dyslipidemia, Heart disease	2.322	82.888	1.982
Hypertension	Dyslipidemia, Diabetes	1.763	82.394	1.971
Hypertension	Dyslipidemia, CVA	1.527	78.862	1.886
Hypertension	Diabetes	9.857	74.307	1.777
Hypertension	Dyslipidemia	5.053	71.499	1.710
Hypertension	CVA, Heart disease	3.849	70.968	1.697
Hypertension	CVA	9.559	64.545	1.544
Hypertension	Heart disease	17.418	63.507	1.519
Heart disease	Dyslipidemia, Diabetes	1.763	62.676	3.598
Heart disease	Dyslipidemia, CVA	1.527	61.789	3.547
Heart disease	CVA, Diabetes	1.825	53.741	3.085
Heart disease	Dyslipidemia, Hypertension	3.613	53.265	3.058
Cluster 3		***1.0***	***50.0***	
Depression	Anxiety	17.033	74.344	2.037
Cluster 4		***1.0***	***50.0***	
Arthritis	Rheumatism or RA, Cataracts	1.601	58.915	4.847
Arthritis	Chronic cholecystitis or cholelithiasis, Cataracts	1.428	53.913	4.436
Cataracts	Glaucoma	2.334	52.660	3.632
Cataracts	Chronic cholecystitis or cholelithiasis, Arthritis	1.515	50.820	3.505
Cataracts	Chronic gastrointestinal ulcers, Arthritis	1.316	50.000	3.448

CVA: Stroke and Cerebrovascular Diseases; RA: Rheumatoid Arthritis
